# *Sporosarcina pasteurii* can clog and strengthen a porous medium mimic

**DOI:** 10.1371/journal.pone.0207489

**Published:** 2018-11-30

**Authors:** Swayamdipta Bhaduri, Carlo Montemagno

**Affiliations:** 1 Ingenuity Lab, Faculty of Engineering, University of Alberta, Edmonton, Alberta, Canada; 2 Department of Mechanical Engineering, University of Alberta, Edmonton, Alberta, Canada; University of Massachusetts Boston, UNITED STATES

## Abstract

The bacterium *Sporosarcina pasteurii* can produce significant volumes of solid precipitation in the presence of specific chemical environments. These solid precipitate particles can enter a network of microscale pores and cause long-range clogging. As a result, the medium gains strength and exhibits superior mechanical properties. This concept is also known as Microbiologically Induced Calcite Precipitation (MICP). In this study, we have used sponge blocks as surrogate porous media mimics and analyzed several aspects of MICP. A synergistic approach involving electron microscopy (SEM), computerized X-Ray tomography (*μ*CT), quasi-static compressive load testing and chemical characterization (EDX) has been used to understand several physical and chemical aspects of MICP.

## 1. Introduction

Using microorganisms like bacteria to seal cracks and plug fractures is a relatively new idea. Some bacteria like *Sporosarcina pasteurii* have the unique capability to secrete large volumes of urease, an enzyme that catalyzes the hydrolysis of urea into simpler organic molecules [1]. Through a series of linked chemical reactions, these bacteria can ultimately lead to the formation of calcium carbonates in aqueous media. This phenomenon is often called Microbiologically Induced Calcite Precipitation (MICP) and has the potential of causing both short and long-range clogging in porous media. MICP has attracted attention in recent years in applications as varied as underground carbon storage and sequestration [2][3], heritage structure conservation [4][5], reservoir engineering [6], high-pressure bore-wells [4][7] and many others.

The existing body of literature in this area focusses primarily on macro-scale systems and the enhancement in their mechanical properties resulting from MICP. These studies do not report on the microscale bacteria-level analysis of the complex interplay between fluid mechanics, solid mechanics and chemical thermodynamics of the precipitation process. Furthermore, a majority of the previous literature on bacterial transport inside porous media deal with packed column experiments, which do not attempt to capture the heterogeneous nature of the bio-hydrodynamics (w.r.t. permeability and porosity) inside real pore networks. It has also been argued [8] that the hydraulic regime inside granular matrices is intrinsically dissimilar to those inside structural fractures and hence, a transfer of transport parameters from one system to another is complicated.

It is now well established that enhancement in strength is possible in porous structural material via microbiological means. Examples of microbes (bacteria) which have recently been popular within the structural engineering community include *S*. *pasteurii*, *B*. *megaterium* and *Shewenella sp*. Especially for MICP caused by *S*. *pasteurii*, the effects of factors like fibre-addition [9], oxygen availability [10], concentrations of cells, enzymes and cementation media [11] have been well-documented. Many different studies have provided evidence of MICP-mediated strength enhancement within a wide range of engineering materials.

In a set of experiments with specimens of cement mortar paste containing various ratios of fly ash and *B*. *megaterium* cells, the specimens were subjected to a post-treatment with a mixture of nutrient broth urea (NBU) enriched with CaCl_2_ [12]. Compared to control samples, these exhibited superior compressive strength. Although there was no significant improvement after a week, the strength increased by more than 20% in four weeks. Another study involving *Shewenella sp*. extracted from river waters and mixed with mortar paste showed definitive increase in strength at concentrations higher than 10^5^ CFU/mL. This measurement was further complemented with imaging studies showing the existence of fibrous structures inside the porous network that reduce the effective porosity [13]. A clever study encapsulated *S*. *pasteurii* cells inside Siran^TM^ glass beads [14]. Concrete bars with artificially indented cracks of uniform dimensions on them were used as specimens. The control samples had their cracks filled with empty beads, nutrient solution and CaCl_2_. The other set had, in addition to the above, live cells of *S*. *pasteurii* injected onto the cracks. This set showed better strength for all concentrations (107–10^9^ cells/cm^3^) after a week and a month. It may be noted that the glass beads immobilized with bacteria provided additional surface area for the formation of a calcite layer that served as a protective coating on the cement, this increasing durability.

In the present work, commercial sponge blocks have been used as porous media mimics. A holistic understanding of the process from its physical, chemical and biological perspectives has been attempted by performing comprehensive examinations of several mechanistic aspects such as compressive strength, pore-scale geometry (via *μ*-CT scanning technology) as well as electron microscopy visualizations at the length scale of the pore-network. A critical challenge surrounding all MICP-based technologies is the prevention of enhanced cementation in the immediate neighborhood of the inlet, an artifact of the inherent non-uniform distribution of bacteria inside a micro-scale network. In this respect too, our study complements the pre-existing breakthrough-curve based analyses in the literature.

Although it’s possible to construct relatively simple network geometries via machining, glass etching or lithography; fabrication of complex 3-D pore systems that resemble a natural fracture is extremely difficult [15]. This is the primary reason why off-the-shelf commercial sponge blocks have been used as experimental specimens. The typical sponge block has a range of characteristic length scales in the form of variable pore-size and geometry that is similar to a real inhomogeneous porous medium like a geologic structure, rock, sandstone etc. The objective of the present work is to characterize the sponge samples used as test samples and to examine the extent of enhancement in mechanical properties post-treatment by pore blockage from the bacteria-induced chemical precipitation. The samples have been analyzed before and after treatment with respect to multiple factors.

There have been a few previous studies on *S*. *pasteurii* or other bacteria and their roles in enzyme-mediated ureolysis/calcite precipitation inside porous media. Most of these studies have focused on large-scale systems and modifications in bulk properties. These studies have not concentrated on the smaller length scales of the order of microns and none have applied a combination of tools like x-ray imaging and scanning technology to quantify systemic parameters.

For example, the possibility of using *S*. *pasteurii* to mitigate potential CO_2_-leakage from underground storage reservoirs has been explored before [16]. It is argued that low-viscosity fluids like biofilms are advantageous in this context given they reduce the required injection pressures and increase the radius of influence around the point of injection. It has been possible to design a strategy to uniformly distribute biofilm-induced calcite precipitation inside a sand-filled column and also to seal a Boyles sandstone core which had been hydraulically fractured. A 2–4 orders of magnitude reduction in permeability and a tripling of tolerance for bore pressure were reported as results. Tracer imaging and time-lapse photography have also been used to determine the spatio-temporal distribution of calcite crystals [17]. This study investigated the efficiency of *S*. *pasteurii*—induced MICP inside small and large-scale artificial fractures consisting of a rough rock lower surface and a smooth plastic upper surface. A modified injection strategy was designed to accelerate the process as compared to the continuous flow arrangement. It was also possible to control the extent of precipitation by regulating the injection fluid velocity.

One main novelty of the present work is the application of high-resolution x-ray imaging and computerized tomography tools to visualize the pore network and understand the three-dimensional nature of the pore geometry. There exist some examples of similar studies in non-biological contexts.

Nonetheless, the geometric aspects of an entire pore network has been previously compared with individual fractures in the network [18]. In a natural coal sample, a combination of Wood’s metal injection technique with X-ray Computerized Tomography (CT) was used to analyze the aperture distribution. The distribution was found to be anisotropic and a function of individual pore geometry. A 3-D autocorrelation analysis and a 2-D planar analysis were performed respectively at the network and individual fracture levels. An integrated system to measure and analyze a fracture network was subsequently developed [19]. It could correlate network geometry with physical properties of the system by using a hybrid approach involving X-ray tomography and 3-D image processing to visualize the fracture geometry in a coal core sample. The variation in porosity as a function of displacement along the core axes could be quantified as well.

Elsewhere, a high-density high-contrast metal injection technique has been combined with gravimetric analysis and tomographic imaging under lithostatic conditions to reconstruct the fracture topology with one micron resolution [20]. The spatial quantifications on the distribution of pore sizes and connectivity inside an opaque bituminous coal sample were also obtained. In yet another study [21], the distribution of network porosity in core rock samples containing natural fractures was analyzed. Medical imaging algorithms were combined with Wood’s metal injection technique to compute the mean network aperture over the length scale of the core and to analyze the associated 3-D geometry. Graph theory was applied to the reconstructed network to determine transport parameters like the capillary pressure-saturation relationships. X-ray imaging has also been used on bio-cemented Ottawa 50–70 sand samples before and after triaxial compression tests to observe an abrupt change in the mechanism of deformation from homogeneous to dilatant shearing at peak stress [22].

A different paradigm in scanning technology, the magnetic resonance imaging (MRI) has also been used to study bacterial systems. One approach used a combination of MRI and immuno-magnetic labeling to non-invasively visualize the changes in spatial distribution of a bacteria population inside a saturated porous medium [23]. They attached magnetite particles on the cell surface of *E*. *coli* to track the movements of individual cells and compared the observations with existing transport models for motile and non-motile biological cells. Very recently, MRI and *μ*-CT were combined as complementary techniques to quantify the advective and dispersive aspects of chemical precipitation inside a porous medium [24]. Although the study did not use bacteria, this technique can well be extended to any reactive transport system such as MICP. MRI in conjugation with Nuclear Magnetic Resonance (NMR) imaging has also been used to study the process of MICP inside a model glass bead pack and a real sandstone core [25]. Precipitation was seen to occur mostly near the inlet for the model bead system whereas it was more uniform for the natural system. Another study discusses the problem of fluid transport inside a polyurethane foam and the effects of compression on the velocity field using MRI [26].

There also exists a large body of literature on the physical, chemical and biological aspects of MICP. The present work not only complements but builds up on many previous findings. A few must be mentioned here. *P*. *fluorescens* and *P*. *cepacia* cells labeled with radionuclides have been used to understand the effect of fluid velocity on motile and non-motile bacterial transport inside saturated porous media [27]. This work extended the colloid filtration theory to examine the effects of fluid flow on cell retention rates and observed some deviations from theoretical predictions. An effort to introduce additional parameters to account for the discrepancy and identify an optimum was also made. In a different study, the researchers used saline and phosphate buffer solutions to grow *S*. *pasteurii* and *P*. *aeuruginosa*. Both live and dead cells were used for comparison. It was found that saline buffers are detrimental to increase of strength. This observation was explained using ionic equilibrium arguments resulting form addition of Cl^-^ ions [28].

The general physical and chemical variables influencing microbial transport inside porous media have also been studied [29]. Effects of several variables like ionic strength, cell size, grain size and heterogeneity of the media on cellular transport were quantified by adding resting-cell suspensions of bacteria on top of packed clean quartz sand columns and pumping artificial groundwater all the way through. The change in permeability of a porous medium resulting from in situ enzymatic production of calcium carbonate has been investigated as well [30]. No bacteria were used for their experiments. Effects of enzyme concentration, temperature and amount of reactants (urea and calcium chloride) too were studied on a batch system of Berea sandstone cores and unconsolidated porous media. It was examined how multiple injections affect the efficiency of plugging.

The possibility of extending random walk calculations originally developed for abiotic disordered continuum systems to the problem of bacterial motility in porous media has also been explored [31]. Model porous media were generated using molecular dynamic simulations and the Einstein relation was applied to compute the effective bacterial diffusion coefficient. Yet another new idea that has been explored is that of autogeneous healing via MICP. In contrast to the usual approach where the bacterial agent is supplied externally, this technique involves integrating active cells in the construction material (cement, mortar, concrete) under application. It is proposed that self-integrated microbial populations are more efficient than external populations in achieving MICP-based bioremediation (sealing cracks and plugging holes) under a wider range of conditions. The study involving *B*. *cohnii* cells integrated onto coarse concrete is worth referencing [32].

Last but not the least, there have also been instances of results that question the effectiveness of MICP-based strengthening techniques. A comparative study [33] of the 3-point compressive and bending strengths of concrete slabs containing four different bacterial strains (*S*. *pasteurii*, *B*. *pseudofirmus*, *B*. *halodurnas* and *B*. *cohnii*) with control samples failed to measure any improvements. At least one piece of literature casts a doubt on the capability of *S*. *pasteurii* to enhance mechanical strength via MICP. This study investigated the effects of five calcite-forming bacteria (CFB) species including *S*. *pasteurii* on mortar strength [34]. For the experiments, a fresh inoculum was cultured to be mixed with the mortar, which was subsequently cured in a solution of urea and CaCl_2_. The other four species under investigation were: *S*. *soli*, *B*. *massiliensis*, *A*. *crystallopoietes* and *L*. *fusiformis*. Only two out of the five species (*B*. *massiliensis*, *L*. *fusiformis*) could produce measurable difference in compressive strengths.

## 2. Materials and methods

### 2.1. Compression tests

All compression tests were performed on carefully prepared cuboidal sponge blocks. Each specimen was cut out from a larger sponge block procured directly from commercial vendors (LeadingSponge, Guangdong, China). The average density of each block is reported to be 48 kg/m^3^ and the average spatial void fraction is about 90%. The type of sample chosen was of flexible, open-foam variety. These samples are highly anisotropic with significant variations in mechanical strength along the three axes. All results reported in this work are based on loading tests performed along the 25 mm axis that is the orientation of the foaming. The mechanical behavior for loading along the other two axes has been shown in the supplementary material (Figures J and K in S1 File). The specimens have a tendency to deform in response to applied compressive loads and completely recover back to the initial state once the load is withdrawn. This helps in resisting multiple loading cycles within an yield limit of compressive stress, beyond which the open foam cell walls collapse and completely change the mechanical properties. ASTM standards for open foam cellular material was followed as precisely as possible. A 100-teeth per inch (tpi) industrial saw was used in conjugation with a 3-D printed cutting jig to ensure the planarity of faces. The individual block size was maintained at 100 x 25 x 100 mm^3^.

The untreated control specimens were not subjected to any modification and were tested in their pristine state. A second set of specimens was immersed in the same culture medium which was not inoculated i.e. did not have any bacteria cells. A third set of specimens was immersed in an inoculated medium and left for 120 hours before being withdrawn from the liquid and air dried inside a biosafety hood for 48 hours. The existing protocol for enrichment and acceleration of precipitation was followed, as described in [35].

For our experiments, an Instron 5960-series Dual Column mechanical test unit with compression loading cells (10kN) to load the samples was used. A load cell is a device used to measure weight or force under compressive, tensile, bending or shear loadings. It is essentially a transducer unit which has a membrane stressed to a predetermined magnitude. The membrane is usually connected to metal foil strain gauges that produce an electrical output when subjected to external stress. The electrical signal is amplified and read as a calibrated result and displayed with units of mechanical stress/strain.

A very small loading rate of 1mm/min was selected to approximate quasi-static conditions. A a test termination criterion of 9kN compressive load before the beginning of compression was selected. Computations of compressive stresses, strains, stretch ratios and Young’s Moduli were performed in MATLAB from the load-displacement raw data generated by the machine.

### 2.2. Micro-computed tomography (μCT)

For the second aspect of our experiments, extensive *μ*-CT scans on the specimens were performed. This helped us numerically compute parameters such as porosity and pore connectedness. A comparison of unclogged and clogged samples demonstrated the reduction of porosity post-treatment. The 3-D reconstruction of the clogged samples also gave a clear visual representation of the network topology of pores and the nature of connectedness.

CT stands for computerized tomography. It uses an X-ray source to image a solid volume from many different angles in space and combines all the virtual slices of the specific areas to produce a complete tomography or a cross-sectional image of the body. It thus allows one to see inside an otherwise opaque object without cutting through it physically. Comprehensive reconstruction and volume rendering algorithms are available for generating a 3-dimensional image of the interior of the target specimen being scanned using a large stack of plane 2-dimensional images acquired about a predetermined axis of rotation.

The high-performance Bruker SkyScan 1172 High Resolution Desktop *μ*CT was used for our experiments. The system has a full distortion correction 10 megapixel X-ray camera which can acquire up to 8000 x 8000 pixels in each single slice while going down to as low as 0.5μm in isotropic detail detectability. It also has a dynamically variable acquisition geometry for the shortest scan at any magnification. All analyses were performed using the in-house surface and volume rendering packages (CTAn and CTVol).

### 2.3. Microscopy and chemical analyses

Finely sliced segments of the control and non-control sponge specimens were subjected to SEM imaging and characterization via Electron Dispersive X-ray spectroscopy (EDX). The sample set under examination contained both Au (metallic) and C (non-metallic) sputtered-specimens. Carbon-coated samples allow the advantage of using much higher accelerating voltages (25–30 kV) that lead to better penetration depths, thus facilitating the imaging of deeply buried precipitate fragments. The microscopy unit was a Zeiss EVO equipped with EDX. Most chemical analyses were performed within the *Aztec* commercial package.

## 3. Results and discussions

**Fig 1:**
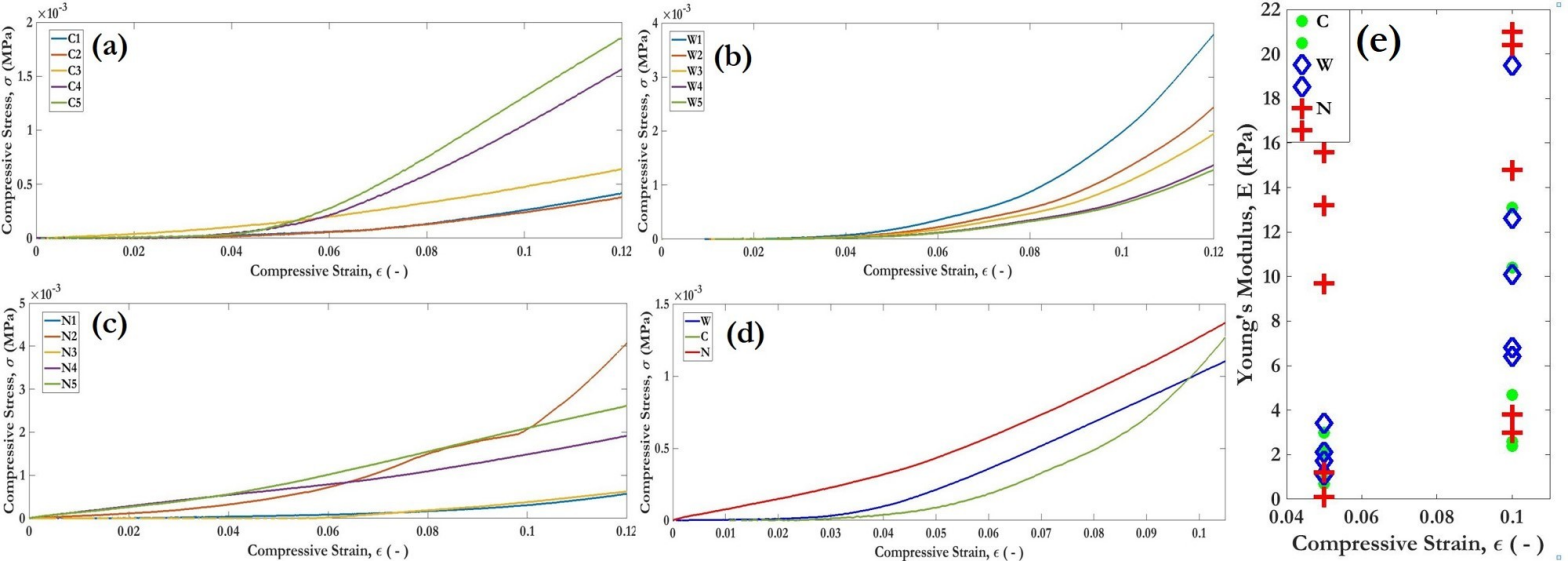
**Stress-strain curves and Young’s Moduli for the test specimens: (a), (b) and (c):Engineering stress as a function of engineering strain for low deformation (ε = 0.1) limit corresponding to C, W and N-sets, (d): superposition of averaged curves from (a)-(c) for direct visual comparison, (e): Scatter Diagram of the Young's Modulus variation at two strain values (0.05, 0.1).** C denotes a control sample which is dry and not treated, N denotes a non-control sample which has been immersed in the bacterial culture and dried before measurements. W is a sample immersed in a non-inoculated culture medium.

The load-displacement data showing the variation of engineering stress as a function of the compression strain has been plotted (Fig 1). It can be seen that there has been a significant enhancement in mechanical strength post-treatment.

All experiments have been restricted to the small deformation (**ε** = 10%) limit. No comparative conclusions have been drawn beyond this limit to ensure the reliability of engineering stress and engineering strain measurements. All stress and strain values reported in this work are pure scalars and simply use the definitions given below:

$$\varepsilon_{Engg.}=\frac{Change\;in\;linear\;dimension\;(mm)}{Initial\;linear\;dimesnion\;(mm)},$$

$$\sigma_{Engg.}=\frac{Normal\;Compressive\;Force\;(N)}{Area\;of\;Cross-section\;(mm^{2})}.$$

For all loading tests, specimens of uniform dimensions have been used. The loading force has been applied along an axis of length 25 mm and normal to a square plane of size 100 x 100 mm^2^. Thus, the initial dimension before the application of loading has always been 25 mm (denominator of **ε**). Similarly, the area of cross-section normal to the loading axis has always been 100 x 100 mm^2^ (denominator of σ). The numerators have been obtained from force-displacement experiments.

Fig 1 shows the stress-strain curve for fifteen different samples. The ones denoted with a **‘C’** are control samples which are dry, never subjected to any treatment and are soft. The ones denoted with an **‘N’** are non-control samples which have been immersed in an inoculum and subsequently dried prior to measurements and have experienced significant hardening. The ones denoted with a ‘**W**’ are a second set of control samples which have been immersed in an uninoculated culture medium (only nutrients sans bacteria cells). All experiments were performed using inoculums with bacterial concentrations of 2.25 x 10^6^ cells/mL, measured using standard CFU (colony forming units) plate counts. It can be clearly seen that **N**-samples demonstrate much higher stress values than **C**-samples at any given strain point. The **W**-samples do show some hardening as compared to the **C**-samples due to wetting and deposition of the constituent particles in the nutrient broth. However, this enhancement is quite smaller than that observed in **N**-samples. In total, five samples have been shown in Fig 1 corresponding to each of the three categories. Fig 1(A), 1(B) and 1(C) correspond to **C**, **W** and **N**, respectively.

Fig 1(D) plots the (pointwise) average of all 5 replicates and superposes them onto a single graph for direct comparison. The color codes for these have been chosen to be green, blue and red, in the same order. Fig 1(E) plots the scatter diagram for Young’s Moduli (E) under compression at two representative strain values of 5% and 10% for all three sets of samples. The maximum E-values at 5% are about 3kPa and 15kPa before and after treatment, respectively (corresponding to **C** and **N**). Similarly, the numbers at 10% are ~ 10kPa and 20kPa, respectively. In terms of arithmetic mean values, there is an enhancement by more than a factor of 5 at 5% strain. The effect of MICP on degree of strength enhancement is much more pronounced at lower strain values like 5% than at higher strain values like 10%. This could be due to several factors other than MICP that cause a hardening of the specimens at high strains e.g. strain hardening due to dislocation and breakdown of the internal structure of the polymer cells and wall collapse. Nonetheless, it can clearly and conclusively be seen that the enhancement in mechanical strength is the direct result of MICP and not an experimental artifact due to wetting or precipitation resulting from the constituent chemicals present in the medium.

**Fig 2:**
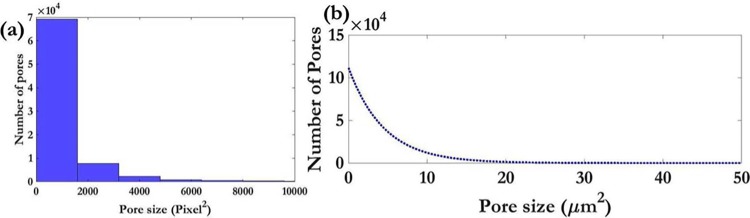
**Analysis of pore network via μ-CT scans**: (a) Histogram of pore sizes in an untreated specimen (b): Probability distribution function of pore sizes in an untreated specimen.

The starting point was the determination of the right representative volume element (RVE) for the 3-D mesh and checked for convergence of porosity, the key parameter under investigation. Once convergence had been ensured, a statistical analysis of the pore size distribution was also performed (Fig 2).

Fig 2 shows the distribution of pore sizes in the sponge blocks. This is a cumulative distribution of individual pore size quantification performed on twelve different samples. Not surprisingly, the pore network has an exponential-type trend where a majority of pores are of the order of 10 μm^2^ or larger. This type of a trend also hints at the presence of an intrinsic randomness inherent in the porous medium. This reinforces the selection of the particular sponge as the porous media mimic of choice because it’s able to partially capture the random nature of a real engineering porous medium like sand or concrete.

**Fig 3:**
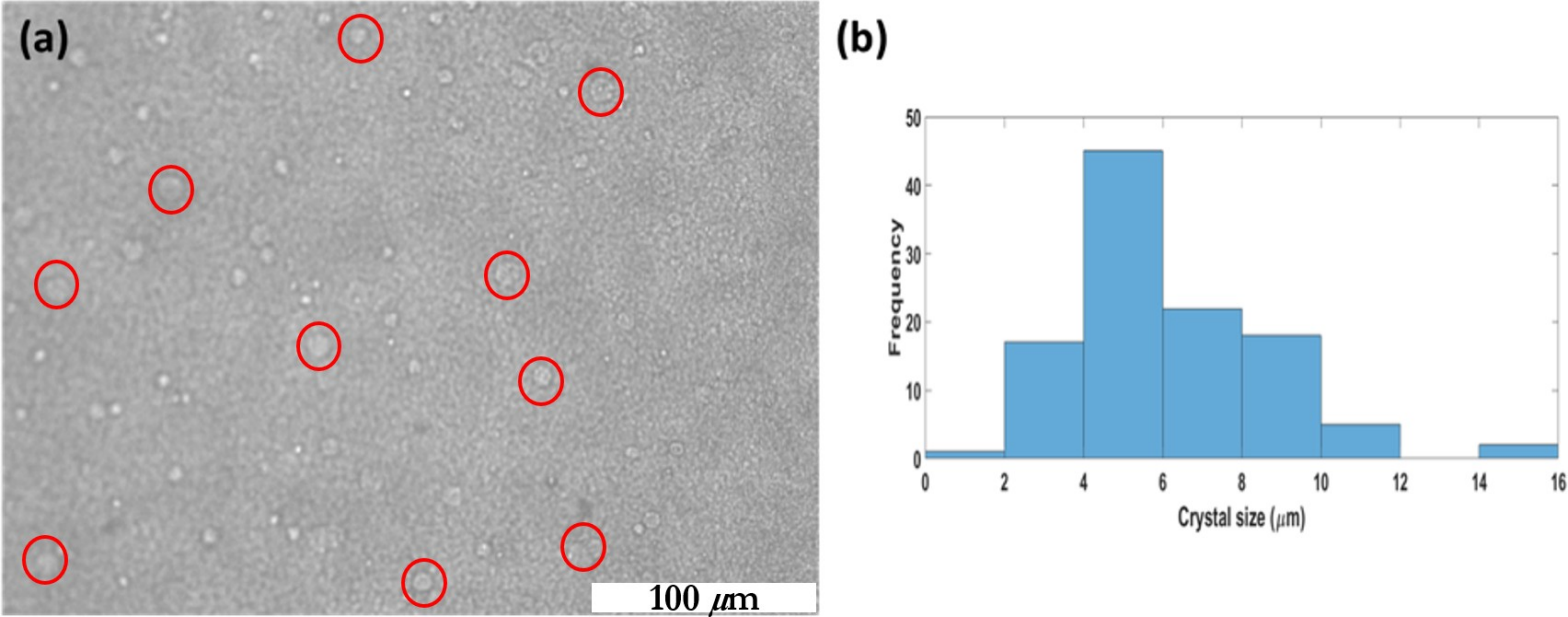
**Analysis of crystal size distribution from a phase-contrast image**: (a) Field of view under analysis (scale bar = 100 μm). (b) Frequency distribution of crystal size distribution in a liquid medium.

It was compared with the distribution of crystal sizes in a liquid culture, extracted form a micrograph via image processing on *ImageJ*. The image processing algorithm adopted for this problem is a standard technique in digital image analysis of particulate matter. The grayscale image obtained from microscopy was pre-processed to enhance contrast and sharpen edges. A calibration of the image magnification was performed to set a length scale associated with a unit pixel. The next step was the determination of thresholds for the image that provide the maximum contrast between the crystal edges and the background. Due to the very nature of the mode of microscopy (phase imaging), the maximum contrast obtained was not of a high quality. It was subsequently decided to subtract a filtered version of the image from itself to obtain a much better contrast. A blurring filter applied to the original image created a copy that mostly contained the background information but not many of the crystals. When this copy was subtracted from the entire image, the resultant image was highly rich in feature (crystal) information. This final version was post-processed by using a brightness enhancement. The enhanced copy was segmented, masked and ultimately used for computation of the crystal size distribution. It can be seen that most crystals can enter most pores which entail a faithful and reliable long-range clogging (Fig 3). Fig 3(A) is a phase-contrast image taken on a stagnant liquid culture prepared on a *Lab Tek*^TM^ II Chambered Coverglass system. The distribution of crystals may be seen as white speckles against the darker background. Fig 3(B) is a histogram of the crystal size distribution which shows that most crystals are about 10μm in size or smaller. When this is compared with the range of pores in a typical porous medium used for our studies, one can clearly see that most crystals would be able to enter most pores. As a result, there would be a penetration that is sufficiently deep to cause sufficient strengthening.

**Fig 4:**
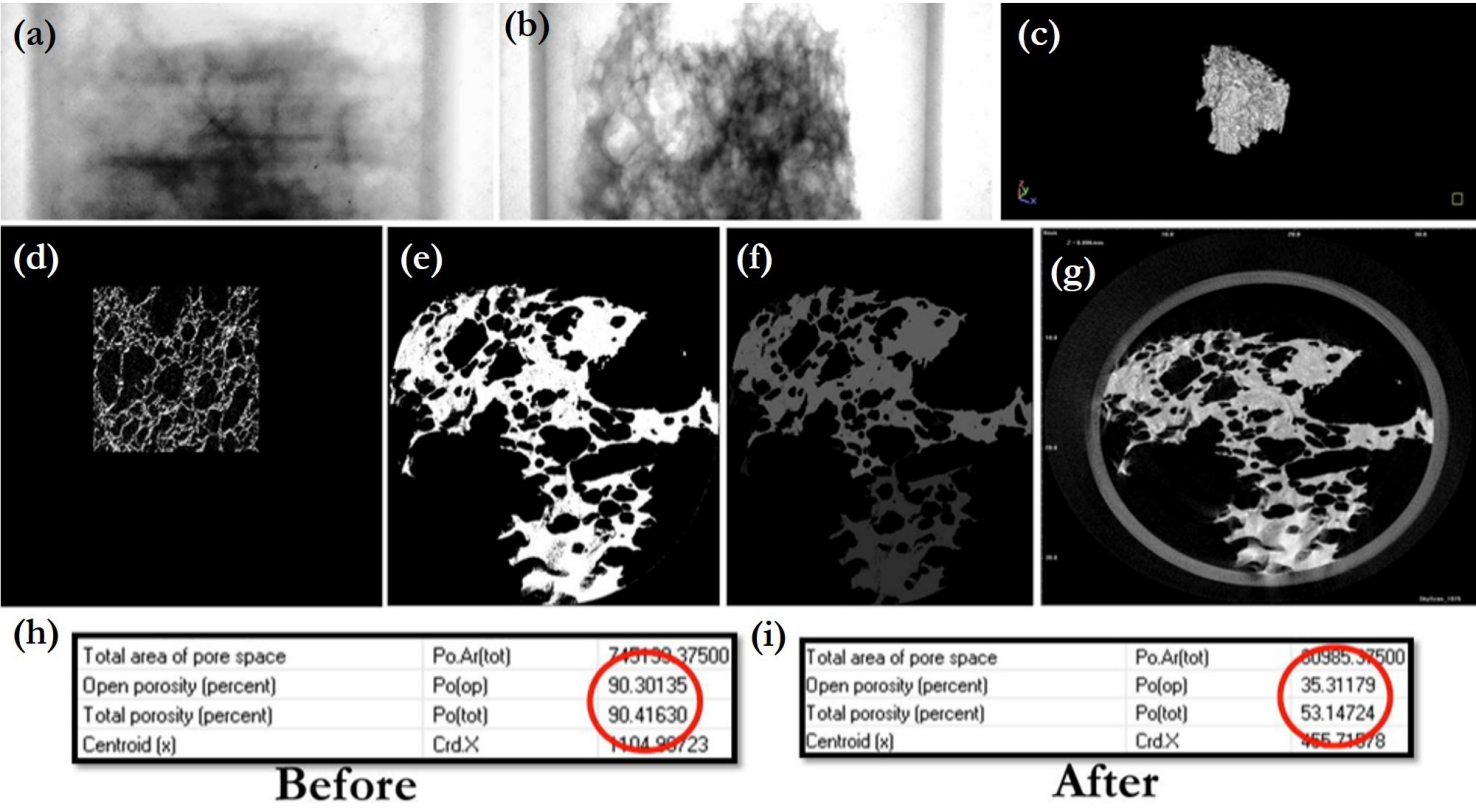
μ-CT investigations of the pore network: Sequence of operations (segmentation, filtration and thresholding) to resolve the different phases in the clogged and unclogged samples. A 3x reduction in porosity has been highlighted.

The tomographical slices were individually color rendered and a full 360^O^ sweep was performed to calculate porosities of unclogged and clogged samples. It was found that there has been a significant reduction in porosity from ~ 90% to about 30%, which correspond to a 3x enhancement in mechanical strength (Fig 4). Fig 4 shows the sequence of steps that have been used to extract quantitative information form the raw scan data files. Fig 4(A) and 4(B) show the raw data obtained from the x-ray camera. Fig 4(C) is a spatial (volumetric) representation of a single sample in 3D. As the specimen rotates, thin slices are imaged for subsequent reconstruction and analysis. Fig 4(D) shows the dorsal section of a sponge sample imaged from behind. The various shades of gray in the grayscale image constitute the “depth” of the image which is in direct correlation with the degrees of attenuation of the incident x-rays by the material, and by extension, several mechanical properties like softness. Fig 4(E) shows a single slice out of many (360 x 2 for images at ½ ^O^ interval per cycle) which add up to form the complete stack. Fig 4(F) is a transformation of 4(e) after the application of a watershed-type filter that brings out the finer details of a foreground in contrast to a background. Finally. 4(g) is an azimuthal projection of 4(e) as viewed from a pole of the spherical sweep space of image acquisition. The raw data in the native proprietary format must be suitably enhanced and successively filtered before it is subjected to voxel thresholding for resolving the three different phases in three dimensions: the void pores which were not accessible to the precipitated particles, the precipitate particles themselves and the material of the porous medium (sponge) itself. This can be computed only when a suitable definition of pore boundaries has been incorporated into the image processing routine and a numerical distinction between pore space and pore connections (throats and necks) been made. This definition was included *ab initio* through externally developed codes in MATLAB^TM^, used in conjugation with the commercial program available with the hardware. The last step is to do the calculations of the parameters of interest like porosity (Fig 4H and 4I).

**Fig 5:**
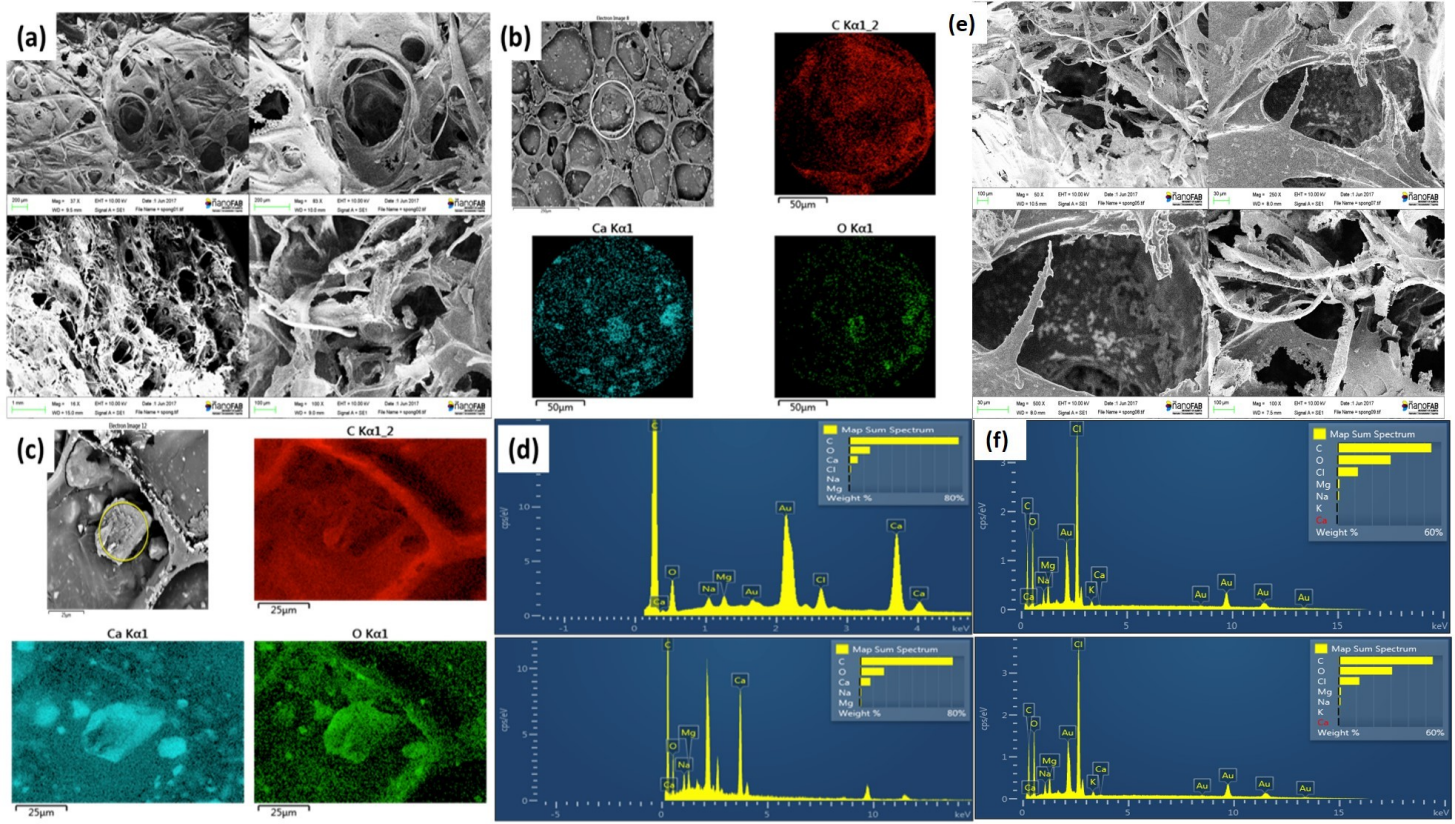
SEM and EDX of the porous medium matrix and the precipitated crystals. (a) Structure of the pore network as imaged under SEM. (b)-(c): Targeted chemical characterization on two pin-pointed locations via EDX. (d) Elemental maps corresponding to (b) and (c) confirming the presence of the signature elements in calcite. (e) The pore network for a similar set of samples immersed in culture medium minus cells. No precipitation detected. (f) Elemental maps show the absence of Ca. The Cl-signals originate from the NaCl in the culture medium. The Au-signals originate from the sputtered gold layer on the sample.

The pore network surface architecture was observed under SEM. For samples treated with the inoculated bacteria culture, conspicuous precipitation can be seen. Targeted chemical signature ID on the precipitated solids was performed with an EDX probe and elemental maps that show the presence and distribution of the chemicals expected from MICP (viz. Calcium, Carbon and Oxygen) were constructed (Fig 5). Gold (Au) was present by virtue of the thin conductive coating deposited by sputtering.

This characterization data provides a conclusive evidence of the actual chemical nature of the precipitates.

## 4. Conclusion

The present work has utilized sponge blocks as surrogate porous medium mimics to study the phenomenon of MICP. This approach has several advantages in terms of the ease of experimentation and the analysis of the various physical, chemical and biological aspects associated with the process. Through techniques such as μ-CT scanning and SEM, we have tried to understand the nature and extent of precipitation inside the real pore network and also correlated precipitation with enhancement in mechanical strength. This is the first study that quantifies the phenomenon of MICP inside micro-scale pores with a synergy of compressive strength, computerized micro-tomography and porosity data.

The first key observation is the inherent non-linearity in the mechanical response of the sponge samples. Being open-celled foams, they exhibit several deformational degrees of freedom per each loading axis. This is manifested in the distribution of compression moduli under low and high shear stresses. Although there is a spread, a significant enhancement is mechanical strength is observed in a statistical sense. For an extended porous medium, where arbitrarily chosen small volumes may be analogous to individual sponge blocks, a statistical increase in compressive strength would add up incrementally, giving rise to an overall increase in strength.

Another important aspect of the results is the quantification of pore connectedness through the 3D reconstruction of the network topology and the associated comparison in porosity. For a large-scale application that’s targeted towards end-uses like underground carbon storage for long time-scales, this is of especial interest to the success of the technology. Limiting the available transport footprint of the fluids stored under high pressure by offering a lower porosity network path not only increases the stability of the storage system, but also ensures greater reliability and safety of operation. The micro-tomography results obtained in the present work may be extended to field-scale industrial applications to gain deeper insights into clogging process at the dimensions of microns or even smaller.

Finally, another important aspect of the present research must be mentioned. The primary motivation behind the problem was the possibility of extending the idea of bacteria-mediated pore clogging to real-life large-scale engineering systems like underground carbon sequestration reservoirs or concrete structures. Although the present findings look quite promising, caution must be exercised while directly extrapolating the results for macroscopic length scales. All of the experimental results reported here have been based on microfluidic and microscale platforms. The culture conditions have been ideal laboratory-based competition and only a single species (*S*. *pasteurii*) of bacterium has been present all throughout. Additionally, the representative porous media chosen have only a limited capacity to mimic the physical and chemical properties of a wide range of engineering materials. In contrast, any technique that successfully extends this idea to a reliable and repeatable sealing method for cracks and fractures, must take into account the biological competition from other species present in any natural non-lab ecosystem. It should also have the versatility to be implemented across a large variety of materials like rocks, sands, clays etc. This can only be achieved through more in-depth experimental analyses and carefully designed parametric studies. Much more needs to be done before a comprehensive understanding of MICP emerges. Effects of several critical real-life factors like surface chemistry, material properties of the porous medium itself and other biological factors still remain less clearly understood.

## Supporting information

S1 File Figure A. SEM image performed on the surface of a specimen. Deposits of white precipitate particles can clearly be seen within the pore wall boundaries.
Figure B. Targeted EDX performed on the same sample as shown in Figure A above. Individual chemical IDs corresponding to Ca, C and O confirm the existence of the three elements produced via MICP.
Table A. Various elements identified within the solid precipitates as imaged in Figure A.
Table B. Dispersive spectroscopic information for the characterization corresponding to Table A above.
Figure C. High magnification image of precipitated crystals.
Figure D. EDX spectrum corresponding to Figure C above.
Table C. Various elements identified within the solid precipitates as imaged in Figure C.
Table D. Dispersive spectroscopic information for the characterization corresponding to Table C above.
Figure E. Targeted EDX performed on the same sample as shown in Figure C above. Individual chemical IDs corresponding to Ca, C and O confirm the existence of the three elements produced via MICP.
Table E. EDX detector system parameters corresponding to Figure D.
Table F. Back-scattered electron spectroscopy data acquisition parameters corresponding to Figure C.
Figure F. Single crystals trapped within the walls of a pore.
Figure G. EDX spectrum corresponding to Figure F above.
Table G. Various elements identified within the solid precipitates as imaged in Figure F.
Table H. Dispersive spectroscopic information for the characterization corresponding to Table G above.
Table I. EDX detector system parameters corresponding to Figure G.
Figure H. Targeted EDX performed on yet another sponge specimen. Individual chemical IDs corresponding to Ca, C and O confirm the existence of the three elements produced via MICP.
Figure I. EDX spectrum corresponding to Figure H above.
Table J. Various elements identified within the solid precipitates as imaged in Figure H.
Table K. Dispersive spectroscopic information for the characterization corresponding to Table J above.
Figure J. Load-displacement curve for loading along the *x*-axis.
Figure K. Load-displacement curve for loading along the *y*-axis.
Figure L. The compression test-rig.
(DOCX)

S1 Dataset Raw data. (ZIP)
