# Supplementary material for: Sporosarcina pasteurii can clog and strengthen a porous medium mimic
Source: PLoS One. 2018 Nov 30;13(11):e0207489. doi: 10.1371/journal.pone.0207489 (PMC6267956; doi:10.1371/journal.pone.0207489)
Supplement: S1 File — Figure A. SEM image performed on the surface of a specimen. Deposits of white precipitate particles can clearly be seen within the pore wall boundaries. Figure B. Targeted EDX performed on the same sample as shown in Figure A above. Individual chemical IDs corresponding to Ca, C and O confirm the existence of the three elements produced via MICP. Table A. Various elements identified within the solid precipitates as imaged in Figure A. Table B. Dispersive spectroscopic information for the characterization corresponding to Table A above. Figure C. High magnification image of precipitated crystals. Figure D. EDX spectrum corresponding to Figure C above. Table C. Various elements identified within the solid precipitates as imaged in Figure C. Table D. Dispersive spectroscopic information for the characterization corresponding to Table C above. Figure E. Targeted EDX performed on the same sample as shown in Figure C above. Individual chemical IDs corresponding to Ca, C and O confirm the existence of the three elements produced via MICP. Table E. EDX detector system parameters corresponding to Figure D. Table F. Back-scattered electron spectroscopy data acquisition parameters corresponding to Figure C. Figure F. Single crystals trapped within the walls of a pore. Figure G. EDX spectrum corresponding to Figure F above. Table G. Various elements identified within the solid precipitates as imaged in Figure F. Table H. Dispersive spectroscopic information for the characterization corresponding to Table G above. Table I. EDX detector system parameters corresponding to Figure G. Figure H. Targeted EDX performed on yet another sponge specimen. Individual chemical IDs corresponding to Ca, C and O confirm the existence of the three elements produced via MICP. Figure I. EDX spectrum corresponding to Figure H above. Table J. Various elements identified within the solid precipitates as imaged in Figure H. Table K. Dispersive spectroscopic information for the characterization correspo [file pone.0207489.s001.docx]

**Supporting Information**

**Section A.** **Chemical characterization of the precipitates inside the porous media**

The sponge specimens were subjected to detailed chemical analyses post-precipitation. Thin sections were cut out from the standard sponge blocks used for compression tests and imaged under SEM. The images clearly show the existence of solid precipitated particles entrapped within the pore matrix. This imaging was coupled with targeted EDX scans to pinpoint the signatures of individual elements expected as the end-products of MICP.





Figure A above shows the surface pore network architecture. Big pores lead to small pores and small pores lead to even smaller pores, thus creating a hierarchy of various sizes within the pore system. This network can be accessed through the tiny openings visible on the surface.

Coexisting are white crystals of solid precipitates. Some small crystals may even be seen being trapped inside some of the smaller pores located at slightly deeper depths.


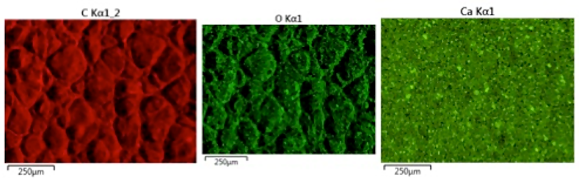


In Figure B above, three different panels correspond to three different chemical signatures of Ca, C and O. On the image that corresponds to Ca, the bright speckles closely correlate to the actual distribution of the solid crystals. The other two (C and O) show a more uniform illumination pattern. This provides us with strong positive evidence about the nature of the chemical precipitation.

| Element | Line Type | Apparent Concentration | k Ratio | Wt% | Wt% Sigma | Standard Label | Factory Standard |
| --- | --- | --- | --- | --- | --- | --- | --- |
| C | K series | 32.79 | 0.32793 | 77.28 | 0.14 | C Vit | Yes |
| Ca | K series | 7.57 | 0.06764 | 5.98 | 0.03 | Wollastonite | Yes |
| O | K series | 4.16 | 0.01399 | 14.59 | 0.14 | SiO_2_ | Yes |
| Cl | K series | 1.36 | 0.01191 | 1.13 | 0.01 | NaCl | Yes |
| Na | K series | 0.61 | 0.00258 | 0.55 | 0.02 | Albite | Yes |
| Total: |  |  |  | 100.00 |  |  |  |

Table A. Various elements identified within the solid precipitates as imaged in Figure A.

Table A shows all the individual elements found in the sample. Ca, C and O are the usual suspects. Na and Mg are present in very low concentrations that are very likely contaminations in Ca. Chlorine shows up as a fixation artifact due to the sample preparation process.

| Element | Line Type | Quant | Area | Sigma | Fit Index |
| --- | --- | --- | --- | --- | --- |
| C | K series | Yes | 403731.58 | 1112.40 | 1514.22 |
| Ca | K series | Yes | 170552.70 | 742.07 | 4.22 |
| Ca | L series | No | -3352.91 | 793.01 | 1162.57 |
| O | K series | Yes | 35502.21 | 388.82 | 326.82 |
| Au | L series | No | 55714.13 | 596.02 | 5.53 |
| Au | M series | No | 262554.66 | 1624.96 | 18.72 |
|  | Noise 1 | No | 148417.51 | 3065.60 | 56.27 |
|  | Noise 2 | No | -173997.32 | 5509.53 | 54.28 |
|  | Noise 3 | No | 98041.61 | 2861.82 | 53.97 |

Table B. Dispersive spectroscopic information for the characterization corresponding to Table A above

Table B provides detailed spectroscopic information on the key elements identified by the characterization process. Ca, C and O are expected elements. Au is an artifact present due to the gold-sputtering process that must be completed prior to the SEM.





Figure C shows a big chunk of solid precipitate. Also visible are smaller grains near the periphery.





Figure D shows the spectrum corresponding to a targeted ID performed on Figure C. Ca, C and O are clearly present. Trace amounts of Mg and Na have also been detected and these are attributed to impurities.

| Element | Line Type | Apparent Concentration | k Ratio | Wt% | Wt% Sigma | Standard Label | Factory Standard | Standard Calibration Date |
| --- | --- | --- | --- | --- | --- | --- | --- | --- |
| C | K series | 18.66 | 0.18661 | 58.76 | 0.15 | C Vit | Yes |  |
| O | K series | 7.35 | 0.02475 | 25.56 | 0.16 | SiO_2_ | Yes |  |
| Ca | K series | 12.30 | 0.10994 | 11.00 | 0.05 | Wollastonite | Yes |  |
| Total: |  |  |  | 100.00 |  |  |  |  |

Table C. Various elements identified within the solid precipitates as imaged in Figure C

| Element | Line Type | Quant | Area | Sigma | Fit Index |
| --- | --- | --- | --- | --- | --- |
| C | K series | Yes | 229747.62 | 890.59 | 860.58 |
| O | K series | Yes | 62790.98 | 493.07 | 187.96 |
| Ca | K series | Yes | 277210.51 | 911.27 | 6.67 |
| Ca | L series | No | -5613.27 | 801.36 | 679.87 |
| Au | L series | No | 61632.84 | 630.37 | 4.70 |
| Au | M series | No | 308486.58 | 1724.68 | 908.16 |
|  | Noise 1 | No | 130271.46 | 2863.93 | 14.44 |
|  | Noise 2 | No | -147771.86 | 5150.02 | 13.92 |
|  | Noise 3 | No | 84389.92 | 2674.28 | 12.95 |

Table D. Dispersive spectroscopic information for the characterization corresponding to Table C above

Tables C and D provide the pertinent information related to the characterization performed on the larger chunk of solid shown in Figure C. As earlier, gold atoms are present as a thin layer of coating on the SEM stubs and hence get picked up as Au peak signals in the spectrograph.


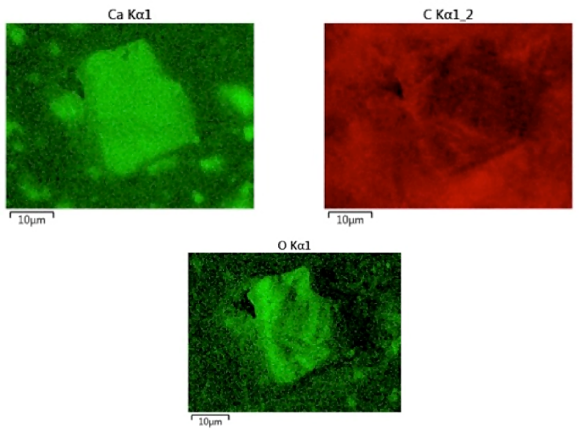


Figure E shows the three individual EDX channels corresponding to the elemental signals for Ca, C and O. The C channel shows homogeneous carpet coverage unlike the two others due to the existence of carbon pigments in the sponge sample.

| Label: | Map Sum Spectrum |
| --- | --- |
| Source: | Acquired |
| Created: | 5/19/2017 11:26:23 AM |
| Livetime: | 195.0s |
| Process Time: | 5 |
| Accelerating Voltage: | 20.00kV |
| Magnification: | 2052 x |
| Working Distance: | 8.4mm |
| Specimen Tilt (degrees): | 0.0 |
| Elevation (degrees): | 35.0 |
| Azimuth (degrees): | 0.0 |
| Number Of Channels: | 2048 |
| Energy Range (keV): | 20 keV |
| Energy per Channel (eV): | 10.0eV |
| Detector Type Id: | 28 |
| Detector Type: | X-Max |
| Window Type: | SATW |
| Pulse Pile Up Correction: | Succeeded |
| Primary Detector: | EDS 1 |
| Primary Detector Serial Number: | 37456 |

Table E. EDX detector system parameters corresponding to Figure D

Table E gives all the relevant information related to the EDX detector used in conjugation with the SEM system. All imaging was performed under an accelerating voltage of 20 kV. This is a relatively low value adjusted so to prevent burn damage on the sample. The gold coating enhances conductivity and facilitates high-contrast imaging under these operating voltages.

| Label: | Electron Image 5 |
| --- | --- |
| Collected: | 5/19/2017 11:50:29 AM |
| Input Signal: | BSE |
| Resolution (Width): | 1024 pixels |
| Resolution (Height): | 768 pixels |
| Image Width: | 770μm |
| Image Height: | 577μm |
| Stage Tilt Degrees: | 0.00° |
| Specimen Tilt Degrees: | 0.00° |
| Software Tilt Correction: | Not applied |
| Magnification: | 153 x |
| Number of Averaged Frames: | 1 |
| Dwell Time: | 20μs |

Table F. Back-scattered electron spectroscopy data acquisition parameters corresponding to Figure C

Table F provides information on the key operational variables related to the image acquisition for Figure C. The image obtained is a high-resolution (1024 x 768) BSE image acquired for 20 μs





Figure F shows a big calcite crystal trapped inside a pore hole. The pore boundaries, which are essentially walled discontinuities between the voids in the sponge, may clearly be seen. There also are smaller grains, which are scattered throughout the area under investigation. Deposits of smaller calcite pigments can be seen everywhere: on the edges, near the boundaries and around the central region.





Figure G shows the EDX graph resulting from the analysis carried out on the sample shown in Figure F. Apart from trace amounts of Mg, Na and K present through impurities; Ca, C and O are strongly detected.

| Element | Line Type | Apparent Concentration | k Ratio | Wt% | Wt% Sigma | Standard Label | Factory Standard | Standard Calibration Date |
| --- | --- | --- | --- | --- | --- | --- | --- | --- |
| C | K series | 31.83 | 0.31831 | 73.43 | 0.15 | C Vit | Yes |  |
| O | K series | 4.26 | 0.01434 | 16.48 | 0.16 | SiO_2_ | Yes |  |
| Ca | K series | 10.06 | 0.08987 | 8.73 | 0.04 | Wollastonite | Yes |  |
| Na | K series | 0.80 | 0.00336 | 0.82 | 0.02 | Albite | Yes |  |
| Mg | K series | 0.42 | 0.00276 | 0.46 | 0.01 | MgO | Yes |  |
| K | K series | 0.08 | 0.00072 | 0.07 | 0.01 | KBr | Yes |  |
| Total: |  |  |  | 100.00 |  |  |  |  |

Table G. Various elements identified within the solid precipitates as imaged in Figure F

| Element | Line Type | Quant | Area | Sigma | Fit Index |
| --- | --- | --- | --- | --- | --- |
| C | K series | Yes | 391885.65 | 1338.71 | 122.26 |
| Ca | K series | Yes | 226608.71 | 835.51 | 2.64 |
| Ca | L series | No | -10508.96 | 819.43 | 103.98 |
| O | K series | Yes | 36373.86 | 399.74 | 52.83 |
| Na | K series | Yes | 14154.09 | 379.22 | 6.60 |
| Mg | K series | Yes | 11979.85 | 380.20 | 24.13 |
| K | K series | Yes | 2025.35 | 333.15 | 519.03 |
| K | L series | No | -100407.25 | 1194.67 | 126.80 |
|  | Noise 1 | No | 213634.89 | 3507.52 | 41.34 |
|  | Noise 2 | No | -262569.61 | 6328.08 | 40.04 |
|  | Noise 3 | No | 142784.37 | 3300.50 | 40.46 |

Table H. Dispersive spectroscopic information for the characterization corresponding to Table G above

| Label: | Map Sum Spectrum |
| --- | --- |
| Source: | Acquired |
| Created: | 5/19/2017 12:13:52 PM |
| Livetime: | 195.0s |
| Process Time: | 5 |
| Accelerating Voltage: | 20.00kV |
| Magnification: | 1026 x |
| Working Distance: | 8.3mm |
| Specimen Tilt (degrees): | 0.0 |
| Elevation (degrees): | 35.0 |
| Azimuth (degrees): | 0.0 |
| Number Of Channels: | 2048 |
| Energy Range (keV): | 20 keV |
| Energy per Channel (eV): | 10.0eV |
| Detector Type Id: | 28 |
| Detector Type: | X-Max |
| Window Type: | SATW |
| Pulse Pile Up Correction: | Succeeded |
| Primary Detector: | EDS 1 |
| Primary Detector Serial Number: | 37456 |

Table I. EDX detector system parameters corresponding to Figure G

Tables G and H show all the important systemic variables present in the analysis of the sample shown in Figure F. All the three compositional elements of calcite (CaCO_3_) namely Ca, C and O remain the major elements detected along with small quantities of Na, Mg and K which enter as impurities of Calcium. Table I accordingly presents the key detector information used in the experiments.


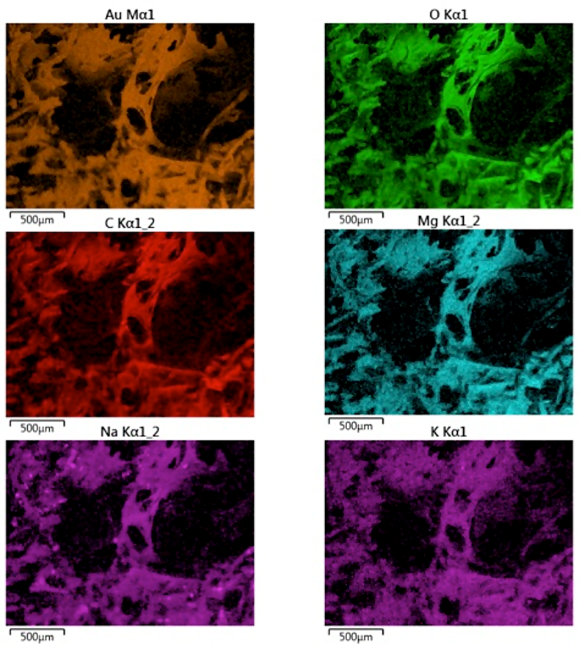


Figure H shows individual channels for one more sample. Au is clearly an artifact of sputtering. The rest are expected results and once again confirm the existence of CaCO_3_.





Figure I shows the spectrogram corresponding to the sample shown above. Apart from Ca, C and O; three extraneous signals have been picked up. Cl is found due to NaCl being used as a fixative. Au originates from the conductive gold plating. Na-Mg-K are impurities present in Ca.

| Element | Line Type | Apparent Concentration | k Ratio | Wt% | Wt% Sigma | Standard Label | Factory Standard | Standard Calibration Date |
| --- | --- | --- | --- | --- | --- | --- | --- | --- |
| C | K series | 1.29 | 0.01289 | 54.24 | 0.54 | C Vit | Yes |  |
| O | K series | 2.19 | 0.00736 | 30.81 | 0.44 | SiO_2_ | Yes |  |
| Mg | K series | 0.24 | 0.00159 | 1.35 | 0.04 | MgO | Yes |  |
| Cl | K series | 2.63 | 0.02295 | 11.98 | 0.15 | NaCl | Yes |  |
| Ca | K series | 0.02 | 0.00022 | 0.11 | 0.03 | Wollastonite | Yes |  |
| Total: |  |  |  | 100.00 |  |  |  |  |

Table J. Various elements identified within the solid precipitates as imaged in Figure H

| Element | Line Type | Quant | Area | Sigma | Fit Index |
| --- | --- | --- | --- | --- | --- |
| C | K series | Yes | 15874.32 | 314.16 | 6.37 |
| O | K series | Yes | 18664.31 | 246.15 | 10.46 |
| Na | K series | Yes | 3632.18 | 155.76 | 0.93 |
| Mg | K series | Yes | 6868.93 | 186.75 | 1.68 |
| Cl | K series | Yes | 78655.39 | 488.79 | 6.17 |
| Cl | L series | No | 700.80 | 121.25 | 18.87 |
| K | K series | Yes | 2287.68 | 151.51 | 2.37 |
| K | L series | No | -4559.48 | 317.07 | 7.73 |
| Ca | K series | Yes | 545.70 | 134.69 | 0.64 |
| Ca | L series | No | -3016.30 | 290.96 | 10.17 |
| Au | L series | No | 17641.58 | 371.40 | 1.16 |
| Au | M series | No | 38259.29 | 646.97 | 5.42 |
|  | Noise 1 | No | 137930.29 | 2566.83 | 24.11 |
|  | Noise 2 | No | -158509.91 | 4671.28 | 23.23 |
|  | Noise 3 | No | 90043.67 | 2448.35 | 24.06 |

Table K. Dispersive spectroscopic information for the characterization corresponding to Table J above.

Tables J and K above provide all the remaining information to complete the characterization picture presented in Figures H and I. This data complements the EDX curves with parametric statistics on spectroscopic and data acquisition aspects of the chemical analysis.

**Section B. Compression tests along the two orthogonal axes**

As described in the main text, we used a 100 x 100 x 25 mm^3^ sponge bar for all our experiments. All data reported were obtained while compressing the sample along the 25 mm (***Z***) axis i.e. the face being compressed was the 100 x 100 mm^2^ (***XY***) plane.

We also performed some tests with the 25 x 100 mm^2^ (***ZX***) plane parallel to the compression platens i.e. loading along the 100 mm (***Y***) axis as well as with the 100 x 25 mm^2^ (***YZ***) face parallel to the platens i.e. compressing along the other 100 mm (***X***) axis.


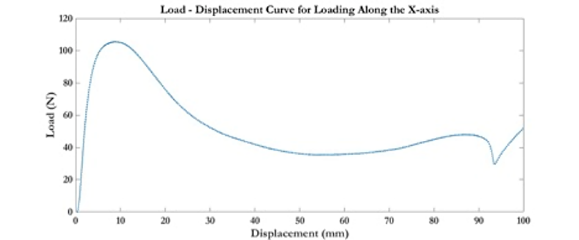


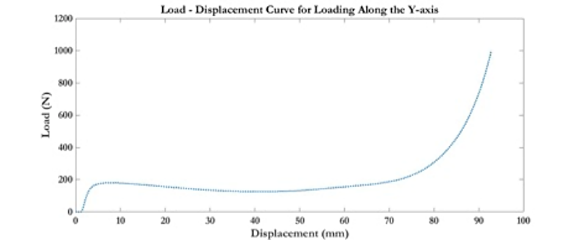


These are shown in Figures J and K above

**Section C. The Compression-test rig**


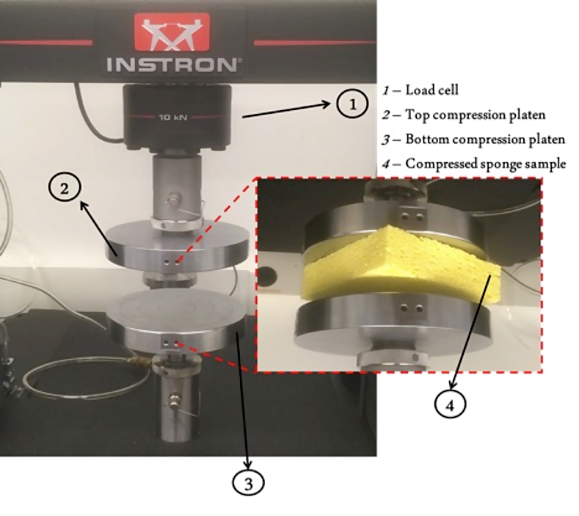


Figure L shows the actual Instron^TM^ universal testing machine. We have used a 10 kN load cell and flat circular compression platens mad of stainless steel. The yellow sponge specimen may be seen being squeezed between the platens. The top platen moves downwards while the bottom platen is stationary. The speed of downward movement of the top platen is regulated to ensure the correctness of the quasi-static approximation.
