# Supplementary material for: Sporosarcina pasteurii can clog and strengthen a porous medium mimic
Source: PLoS One. 2018 Nov 30;13(11):e0207489. doi: 10.1371/journal.pone.0207489 (PMC6267956; doi:10.1371/journal.pone.0207489)
Supplement: S1 Dataset — (ZIP) [file pone.0207489.s002.zip › Raw Data/(for Fig. 4) CT Scans/CT raw data.pdf]

This section provides all the key information related to the micro-CT system, the experimental variables

and the operational parameters.

1 The System
Scanner=Skyscan1076
Instrument SN=13874
Hardware=B
Secure mode=OFF
Software=Version 2. 6 (build 5)
Tube=Hamamatsu 100/250
Camera=Hamamatsu Orca-HRF
Camera Pixel Size (μm)=   11.44
Camera X/Y Ratio=0.9820
2 Data Acquisition
Source Voltage (kV)= 100
Source Current (μA)= 100
Filter=Al 1.0 mm
Object to Source (mm)=121.000
Camera to Source (mm)=161.000
Number Of Rows= 1048
Number Of Columns= 2000
Optical Axis (line)= 400
Image Pixel Size (μm)= 17.2000
Image Format=TIFF
Depth (bits)=16
Data Offset (bytes)= 264
Horizontal overlap (pixel)=0
Camera horizontal position=Center
Visual Camera=OFF
Screen LUT=0
Exposure (ms)= 2360
Rotation Step (deg)=0.500
Frame Averaging=On (3)
Scanning position=88.800 mm
Suggested beam-hardening correction=10
Suggested HU-Calibration=7778
Number of connected scans=1
Use 360 Rotation=NO
Rotation Direction=CC
Scanning Trajectory=ROUND
Type Of Motion=STEP AND SHOOT
Camera Offset=OFF
Scanning Start Angle=196.500
Scan duration=01:01:55
3 Reconstruction statistics

Reconstruction Program=NRecon
Program Version=Version: 1.6.9.18
Reconstruction engine=NReconServer
Engine version=Version: 1.6.9
Reconstruction from batch=No
Postalignment=-2.00
Reconstruction mode=Standard
Dataset Origin=Skyscan1076
First Section=5
Last Section=850
Reconstruction duration per slice (seconds)=1.511820
Total reconstruction time (846 slices) in seconds=1279.000000
Section to Section Step=1
Sections Count=846
Result File Type=BMP
Result File Header Length (bytes)=1134
Result Image Width (pixels)=2000
Result Image Height (pixels)=2000
Pixel Size (μm)=17.20874
Reconstruction Angular Range (deg)=197.00
Use 180+=OFF
Angular Step (deg)=0.5000
Smoothing=0
Ring Artifact Correction=10
Draw Scales=ON
Object Bigger than FOV=OFF
Reconstruction from ROI=OFF
Filter cutoff relative to Nyquisit frequency=100
Filter type=0
Filter type meaning (1)
1: Hann; 2: Ramp; 3: Almost Ramp;
Filter type meaning (2)
11: Cosine; 12: Shepp-Logan; [100,200]: Generalized Hamming, alpha=(iFilter-100)/100
Undersampling factor=1
Threshold for defect pixel mask (%)=0
Beam Hardening Correction (%)=25
CS Static Rotation (deg)=0.00
Minimum for CS to Image Conversion=0.000000
Maximum for CS to Image Conversion=0.026002
HU Calibration=OFF
BMP LUT=0
Cone-beam Angle Horiz.(deg)=16.188761
Cone-beam Angle Vert.(deg)=8.524042

4 Thresholding statistics

Mean (total): 19.759

Selection

Begin: 21
End: 92

Number of voxels: 36547
Mean: 41.399
Standard deviation: 10.548
Standard error of mean: 0.055
95% confidence limits:-
Minimum: 41.289
Maximum: 41.509

5 Analysis

CT Analyser, Version: 1.16.1.0
Operator identity: sbhaduri
Computer name: ALOKE-GRAD03
Computation time 09:10:16
Dataset folder D:\Data\Data\CT Scans\Clogged\Sponge_clogged_Rec\
Pixel size 17.20874 µm
Lower grey threshold: 35
Upper grey threshold: 103

6 Summary (2D data)

Tissue volume, TV 3384000000.00000, pixel^3
Bone volume, BV 295582035.12500, pixel^3
Percent bone volume, BV/TV 8.73469, %
Tissue surface, TS 14767008.84935, pixel^2
Peripheral tissue surface, TS(per) 6767008.84935, pixel^2
Bone surface, BS 82171602.29635, pixel^2
Peripheral bone surface, BS(per) 57307991.29635, pixel^2
Bone surface / volume ratio, BS/BV 0.27800, 1/pixel
Mean total crossectional tissue area, T.Ar 4000000.00000, pixel^2
Mean total crossectional tissue perimeter, T.Pm 7998.82843, pixel
Mean total crossectional bone area, B.Ar 349387.74837, pixel^2
Mean total crossectional bone perimeter, B.Pm 67739.94243, pixel
Mean number of objects per slice, Obj.N 2214.81087, -
Average object area per slice, Av.Obj.Ar 170.42726, pixel^2
Average object area-equivalent circle diameter per slice, Av.Obj.ECDa 14.19053, pixel
Average moment of inertia (x), Av.MMI(x) 37303770416.61176, pixel^4
Average moment of inertia (y), Av.MMI(y) 40435298501.48386 pixel^4
Mean polar moment of inertia, MMI(polar) 77739068918.09547, pixel^4
Average principal moment of inertia (max), Av.MMI(max) 50061278675.04729, pixel^4
Average principal moment of inertia (min), Av.MMI(min) 27677790243.04837, pixel^4
Mean eccentricity, Ecc 0.69435, -
Crossectional thickness, Cs.Th 10.31556, pixel
Trabecular thickness (plate model), Tb.Th(pl) 7.19426, pixel
Trabecular separation (plate model), Tb.Sp(pl) 75.16996, pixel
Trabecular number (plate model), Tb.N(pl) 0.01214, 1/pixel
Trabecular diameter (rod model), Tb.Dm(rd) 14.38852, pixel
Trabecular separation (rod model), Tb.Sp(rd) 28.75716, pixel
Trabecular number (rod model), Tb.N(rd) 0.02318, 1/pixel
Mean trabecular pattern factor, Tb.Pf 0.07013, 1/pixel
Closed porosity (percent), Po(cl) 16.69089, %
Centroid (x), Crd.X 1074.98140, pixel

Centroid (y), Crd.Y 899.94113, pixel
Centroid (z), Crd.Z 338.15223, pixel
Mean fractal dimension, FD 1.48325, -
Total intersection surface, i.S 0.00000, pixel^2
Percent intersection surface, i.S/TS(per) 0.00000, %
Number of layers,,846
Lower vertical position, 5.000,pixel
Upper vertical position, 850.000,pixel
Pixel size, 17.20874,µm
Lower grey threshold, 35
Upper grey threshold, 103
Degree of anisotropy, DA 1.93017 (0.48191)
Eigenvalue 1, 0.46132,
Eigenvalue 2, 0.76854,
Eigenvalue 3, 0.89044,

7 Summary (3D data)

CT Analyzer	Version: 1.14.4.1		
Date and time	15.03.2016 11:51		
Operator identity	skyscan		
Computer name	DOSCHAKCLUSTER0		
Computation time	10:02:05		
Dataset folder	X:\UofA Kumar - Sponge\Sponge_B_clogged\Sponge_clogged_Rec\VOI\		
Pixel size	17.20874	µm	
Lower grey threshold	29		
Upper grey threshold	255		
Total VOI volume	TV	7828.33863	mm^3
Object volume	Obj.V	1616.81819	mm^3
Percent object volume	Obj.V/TV	20.6534	%
Total VOI surface	TS	2338.15417	mm^2
Peripheral VOI surface	TS(per)	1262.72921	mm^2
Object surface	Obj.S	15310.15402	mm^2
Object surface / volume ratio	Obj.S/Obj.V	9.46931	1/mm
Mean total crossectional ROI area	T.Ar	537.71248	mm^2
Mean total crossectional ROI perimeter	T.Pm	86.73427	mm
Mean total crossectional object area	Obj.Ar	111.05592	mm^2
Mean total crossectional object perimeter	Obj.Pm	723.3792	mm
Mean number of objects per slice	Obj.N	507.76359	
Average object area per slice	Av.Obj.Ar	0.29648	mm^2
Average object area-equivalent circle diameter per slice	Av.Obj.ECDa	0.57575	mm
Average moment of inertia (x)	Av.MMI(x)	3407.50666	mm^4
Average moment of inertia (y)	Av.MMI(y)	3685.18746	mm^4
Mean polar moment of inertia	MMI(polar)	7092.69413	mm^4
Average principal moment of inertia (max)	Av.MMI(max)	4645.44981	mm^4

Average principal moment of inertia (min)	Av.MMI(min)	2447.24432	mm^4
Mean eccentricity	Ecc	0.70617	
Crossectional thickness	Cs.Th	0.30705	mm
Structure thickness (plate model)	St.Th(pl)	0.21121	mm
Structure separation (plate model)	St.Sp(pl)	0.81142	mm
Structure linear density (plate model)	St.Li.Dn(pl)	0.97787	1/mm
Structure diameter (rod model)	St.Dm(rd)	0.42242	mm
Structure separation (rod model)	St.Sp(rd)	0.40132	mm
Structure linear density (rod model)	St.Li.Dn(rd)	1.21397	1/mm
Mean surface convexity index	SCv.I	-15.64442	1/mm
Closed porosity (percent)	Po(cl)	22.99809	%
Centroid (x)	Crd.X	13.47657	mm
Centroid (y)	Crd.Y	12.69416	mm
Centroid (z)	Crd.Z	5.94048	mm
Mean fractal dimension	FD	1.49566	
Total intersection surface	i.S	0	mm^2

8 Post-processing

CT Analyser	Version: 1.14.4.1	
Dataset name	G:\UofA Kumar - Sponge\Sponge_B_clogged\Sponge_clogged_Rec\VOI(1)\sponge_clogged_rec_voi_0182.bmp	
File postfix length	4	
File type	BMP	
Image size (W/H)	1271	1053
Total number of images	440	
Total Z-position range	182	621
Number of images inside VOI	440	
Z-position range of VOI	182	621
Z spacing	1	
Pixel size (µm)	17.208742	

Thresholding (3D space)

Mode	Automatic (Otsu method)
Background	Dark

Lower grey threshold	136
Upper grey threshold	255

Thresholding done

Bitwise operations

<Image> = COPY <Region of Interest>

Bitwise operations done

Despeckle

Type: Remove outer objects (3D space)

Detected by: by image borders

Apply to: Region of Interest

Despeckle done

3D model

Model creation algorithm: Marching Cubes 33

Apply to: ROI

Model filename = G:\UofA Kumar - Sponge\Sponge_B_clogged\Sponge_clogged_Rec\VOI(1)\sponge_clogged_rec_voi_.ctm

----

Thresholding (3D space) inside VOI

Mode	Multilevel (Otsu method)
Output color of	Mean intensity in class

class thresholding

Number of threshold levels	2
Level 1	1
Level 2	1
Thresholding done	

2D analysis

Operator identity	sbhaduri	
Computer name	DOSCHAKCLUSTER0	
Computation time	10:00:35	
Dataset folder	G:\UofA Kumar - Sponge\Sponge_B_clogged\Sponge_clogged_Rec\VOI(1)\	
Pixel size	17.20874	µm

9 Histogram from ROI of file

Index (%), Area (pixel^2), Total (%), Selected (%)
0,0.0%,33013.000,40.360%,-
1,0.4%,1214.000,1.484%,-
2,0.8%,993.000,1.214%,-
3,1.2%,940.000,1.149%,-
4,1.6%,945.000,1.155%,-
5,2.0%,813.000,0.994%,-
6,2.4%,753.000,0.921%,-
7,2.7%,624.000,0.763%,-
8,3.1%,609.000,0.745%,-
9,3.5%,573.000,0.701%,-
10,3.9%,505.000,0.617%,-
11,4.3%,470.000,0.575%,-
12,4.7%,485.000,0.593%,-
13,5.1%,434.000,0.531%,-
14,5.5%,460.000,0.562%,-
15,5.9%,433.000,0.529%,-
16,6.3%,377.000,0.461%,-
17,6.7%,397.000,0.485%,-
18,7.1%,385.000,0.471%,-
19,7.5%,402.000,0.491%,-
20,7.8%,424.000,0.518%,-
21,8.2%,424.000,0.518%,1.160%
22,8.6%,484.000,0.592%,1.324%
23,9.0%,507.000,0.620%,1.387%
24,9.4%,477.000,0.583%,1.305%
25,9.8%,584.000,0.714%,1.598%

26,10.2%,590.000,0.721%,1.614%
27,10.6%,696.000,0.851%,1.904%
28,11.0%,732.000,0.895%,2.003%
29,11.4%,758.000,0.927%,2.074%
30,11.8%,809.000,0.989%,2.214%
31,12.2%,878.000,1.073%,2.402%
32,12.5%,919.000,1.124%,2.515%
33,12.9%,1034.000,1.264%,2.829%
34,13.3%,1043.000,1.275%,2.854%
35,13.7%,1177.000,1.439%,3.221%
36,14.1%,1135.000,1.388%,3.106%
37,14.5%,1222.000,1.494%,3.344%
38,14.9%,1231.000,1.505%,3.368%
39,15.3%,1288.000,1.575%,3.524%
40,15.7%,1306.000,1.597%,3.573%
41,16.1%,1292.000,1.580%,3.535%
42,16.5%,1350.000,1.650%,3.694%
43,16.9%,1314.000,1.606%,3.595%
44,17.3%,1321.000,1.615%,3.615%
45,17.6%,1343.000,1.642%,3.675%
46,18.0%,1248.000,1.526%,3.415%
47,18.4%,1177.000,1.439%,3.221%
48,18.8%,1116.000,1.364%,3.054%
49,19.2%,1065.000,1.302%,2.914%
50,19.6%,926.000,1.132%,2.534%
51,20.0%,981.000,1.199%,2.684%
52,20.4%,809.000,0.989%,2.214%
53,20.8%,736.000,0.900%,2.014%
54,21.2%,677.000,0.828%,1.852%
55,21.6%,569.000,0.696%,1.557%
56,22.0%,519.000,0.635%,1.420%
57,22.4%,444.000,0.543%,1.215%
58,22.7%,382.000,0.467%,1.045%
59,23.1%,346.000,0.423%,0.947%
60,23.5%,249.000,0.304%,0.681%
61,23.9%,219.000,0.268%,0.599%
62,24.3%,166.000,0.203%,0.454%
63,24.7%,154.000,0.188%,0.421%
64,25.1%,125.000,0.153%,0.342%
65,25.5%,124.000,0.152%,0.339%
66,25.9%,95.000,0.116%,0.260%
67,26.3%,74.000,0.090%,0.202%
68,26.7%,74.000,0.090%,0.202%
69,27.1%,59.000,0.072%,0.161%
70,27.5%,58.000,0.071%,0.159%
71,27.8%,47.000,0.057%,0.129%
72,28.2%,40.000,0.049%,0.109%
73,28.6%,35.000,0.043%,0.096%

74,29.0%,27.000,0.033%,0.074%
75,29.4%,19.000,0.023%,0.052%
76,29.8%,15.000,0.018%,0.041%
77,30.2%,18.000,0.022%,0.049%
78,30.6%,7.000,0.009%,0.019%
79,31.0%,10.000,0.012%,0.027%
80,31.4%,6.000,0.007%,0.016%
81,31.8%,3.000,0.004%,0.008%
82,32.2%,2.000,0.002%,0.005%
83,32.5%,2.000,0.002%,0.005%
84,32.9%,2.000,0.002%,0.005%
85,33.3%,2.000,0.002%,0.005%
86,33.7%,1.000,0.001%,0.003%
87,34.1%,1.000,0.001%,0.003%
88,34.5%,1.000,0.001%,0.003%
89,34.9%,2.000,0.002%,0.005%
90,35.3%,0.000,0.000%,0.000%
91,35.7%,0.000,0.000%,0.000%
92,36.1%,1.000,0.001%,0.003%
93,36.5%,0.000,0.000%,-
94,36.9%,0.000,0.000%,-
95,37.3%,0.000,0.000%,-
96,37.6%,0.000,0.000%,-
97,38.0%,0.000,0.000%,-
98,38.4%,0.000,0.000%,-
99,38.8%,0.000,0.000%,-
100,39.2%,0.000,0.000%,-
101,39.6%,0.000,0.000%,-
102,40.0%,0.000,0.000%,-
103,40.4%,0.000,0.000%,-
104,40.8%,0.000,0.000%,-
105,41.2%,0.000,0.000%,-
106,41.6%,0.000,0.000%,-
107,42.0%,0.000,0.000%,-
108,42.4%,0.000,0.000%,-
109,42.7%,0.000,0.000%,-
110,43.1%,0.000,0.000%,-
111,43.5%,0.000,0.000%,-
112,43.9%,0.000,0.000%,-
113,44.3%,0.000,0.000%,-
114,44.7%,0.000,0.000%,-
115,45.1%,0.000,0.000%,-
116,45.5%,0.000,0.000%,-
117,45.9%,0.000,0.000%,-
118,46.3%,0.000,0.000%,-
119,46.7%,0.000,0.000%,-
120,47.1%,0.000,0.000%,-
121,47.5%,0.000,0.000%,-

122,47.8%,0.000,0.000%,-
123,48.2%,0.000,0.000%,-
124,48.6%,0.000,0.000%,-
125,49.0%,0.000,0.000%,-
126,49.4%,0.000,0.000%,-
127,49.8%,0.000,0.000%,-
128,50.2%,0.000,0.000%,-
129,50.6%,0.000,0.000%,-
130,51.0%,0.000,0.000%,-
131,51.4%,0.000,0.000%,-
132,51.8%,0.000,0.000%,-
133,52.2%,0.000,0.000%,-
134,52.5%,0.000,0.000%,-
135,52.9%,0.000,0.000%,-
136,53.3%,0.000,0.000%,-
137,53.7%,0.000,0.000%,-
138,54.1%,0.000,0.000%,-
139,54.5%,0.000,0.000%,-
140,54.9%,0.000,0.000%,-
141,55.3%,0.000,0.000%,-
142,55.7%,0.000,0.000%,-
143,56.1%,0.000,0.000%,-
144,56.5%,0.000,0.000%,-
145,56.9%,0.000,0.000%,-
146,57.3%,0.000,0.000%,-
147,57.6%,0.000,0.000%,-
148,58.0%,0.000,0.000%,-
149,58.4%,0.000,0.000%,-
150,58.8%,0.000,0.000%,-
151,59.2%,0.000,0.000%,-
152,59.6%,0.000,0.000%,-
153,60.0%,0.000,0.000%,-
154,60.4%,0.000,0.000%,-
155,60.8%,0.000,0.000%,-
156,61.2%,0.000,0.000%,-
157,61.6%,0.000,0.000%,-
158,62.0%,0.000,0.000%,-
159,62.4%,0.000,0.000%,-
160,62.7%,0.000,0.000%,-
161,63.1%,0.000,0.000%,-
162,63.5%,0.000,0.000%,-
163,63.9%,0.000,0.000%,-
164,64.3%,0.000,0.000%,-
165,64.7%,0.000,0.000%,-
166,65.1%,0.000,0.000%,-
167,65.5%,0.000,0.000%,-
168,65.9%,0.000,0.000%,-
169,66.3%,0.000,0.000%,-

170,66.7%,0.000,0.000%,-
171,67.1%,0.000,0.000%,-
172,67.5%,0.000,0.000%,-
173,67.8%,0.000,0.000%,-
174,68.2%,0.000,0.000%,-
175,68.6%,0.000,0.000%,-
176,69.0%,0.000,0.000%,-
177,69.4%,0.000,0.000%,-
178,69.8%,0.000,0.000%,-
179,70.2%,0.000,0.000%,-
180,70.6%,0.000,0.000%,-
181,71.0%,0.000,0.000%,-
182,71.4%,0.000,0.000%,-
183,71.8%,0.000,0.000%,-
184,72.2%,0.000,0.000%,-
185,72.5%,0.000,0.000%,-
186,72.9%,0.000,0.000%,-
187,73.3%,0.000,0.000%,-
188,73.7%,0.000,0.000%,-
189,74.1%,0.000,0.000%,-
190,74.5%,0.000,0.000%,-
191,74.9%,0.000,0.000%,-
192,75.3%,0.000,0.000%,-
193,75.7%,0.000,0.000%,-
194,76.1%,0.000,0.000%,-
195,76.5%,0.000,0.000%,-
196,76.9%,0.000,0.000%,-
197,77.3%,0.000,0.000%,-
198,77.6%,0.000,0.000%,-
199,78.0%,0.000,0.000%,-
200,78.4%,0.000,0.000%,-
201,78.8%,0.000,0.000%,-
202,79.2%,0.000,0.000%,-
203,79.6%,0.000,0.000%,-
204,80.0%,0.000,0.000%,-
205,80.4%,0.000,0.000%,-
206,80.8%,0.000,0.000%,-
207,81.2%,0.000,0.000%,-
208,81.6%,0.000,0.000%,-
209,82.0%,0.000,0.000%,-
210,82.4%,0.000,0.000%,-
211,82.7%,0.000,0.000%,-
212,83.1%,0.000,0.000%,-
213,83.5%,0.000,0.000%,-
214,83.9%,0.000,0.000%,-
215,84.3%,0.000,0.000%,-
216,84.7%,0.000,0.000%,-
217,85.1%,0.000,0.000%,-

218,85.5%,0.000,0.000%,-
219,85.9%,0.000,0.000%,-
220,86.3%,0.000,0.000%,-
221,86.7%,0.000,0.000%,-
222,87.1%,0.000,0.000%,-
223,87.5%,0.000,0.000%,-
224,87.8%,0.000,0.000%,-
225,88.2%,0.000,0.000%,-
226,88.6%,0.000,0.000%,-
227,89.0%,0.000,0.000%,-
228,89.4%,0.000,0.000%,-
229,89.8%,0.000,0.000%,-
230,90.2%,0.000,0.000%,-
231,90.6%,0.000,0.000%,-
232,91.0%,0.000,0.000%,-
233,91.4%,0.000,0.000%,-
234,91.8%,0.000,0.000%,-
235,92.2%,0.000,0.000%,-
236,92.5%,0.000,0.000%,-
237,92.9%,0.000,0.000%,-
238,93.3%,0.000,0.000%,-
239,93.7%,0.000,0.000%,-
240,94.1%,0.000,0.000%,-
241,94.5%,0.000,0.000%,-
242,94.9%,0.000,0.000%,-
243,95.3%,0.000,0.000%,-
244,95.7%,0.000,0.000%,-
245,96.1%,0.000,0.000%,-
246,96.5%,0.000,0.000%,-
247,96.9%,0.000,0.000%,-
248,97.3%,0.000,0.000%,-
249,97.6%,0.000,0.000%,-
250,98.0%,0.000,0.000%,-
251,98.4%,0.000,0.000%,-
252,98.8%,0.000,0.000%,-
253,99.2%,0.000,0.000%,-
254,99.6%,0.000,0.000%,-
255,100.0%,0.000,0.000%,-
