# Supplementary material for: Sporosarcina pasteurii can clog and strengthen a porous medium mimic
Source: PLoS One. 2018 Nov 30;13(11):e0207489. doi: 10.1371/journal.pone.0207489 (PMC6267956; doi:10.1371/journal.pone.0207489)
Supplement: S1 Dataset — (ZIP) [file pone.0207489.s002.zip › Raw Data/(for Fig. 5) EDX/negative/Project 3_Site 1_2017-06-01_18-42-42.docx]

6/1/2017 6:32:28 PM

Specimen 1


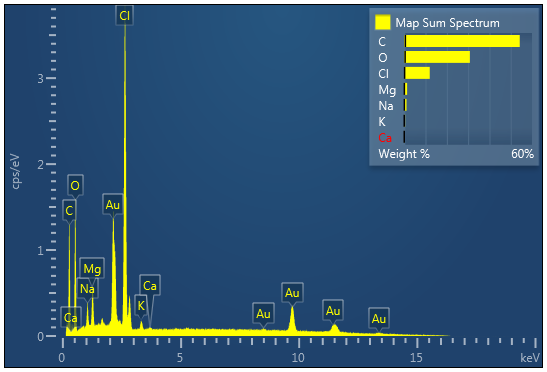


| Element | Line Type | Apparent Concentration | k Ratio | Wt% | Wt% Sigma | Standard Label | Factory Standard | Standard Calibration Date |
| --- | --- | --- | --- | --- | --- | --- | --- | --- |
| C | K series | 1.29 | 0.01289 | 54.24 | 0.54 | C Vit | Yes |  |
| O | K series | 2.19 | 0.00736 | 30.81 | 0.44 | SiO2 | Yes |  |
| Na | K series | 0.20 | 0.00086 | 1.08 | 0.05 | Albite | Yes |  |
| Mg | K series | 0.24 | 0.00159 | 1.35 | 0.04 | MgO | Yes |  |
| Cl | K series | 2.63 | 0.02295 | 11.98 | 0.15 | NaCl | Yes |  |
| K | K series | 0.10 | 0.00081 | 0.43 | 0.03 | KBr | Yes |  |
| Ca | K series | 0.02 | 0.00022 | 0.11 | 0.03 | Wollastonite | Yes |  |
| Total: |  |  |  | 100.00 |  |  |  |  |

| Element | Line Type | Quant | Area | Sigma | Fit Index |
| --- | --- | --- | --- | --- | --- |
| C | K series | Yes | 15874.32 | 314.16 | 6.37 |
| O | K series | Yes | 18664.31 | 246.15 | 10.46 |
| Na | K series | Yes | 3632.18 | 155.76 | 0.93 |
| Mg | K series | Yes | 6868.93 | 186.75 | 1.68 |
| Cl | K series | Yes | 78655.39 | 488.79 | 6.17 |
| Cl | L series | No | 700.80 | 121.25 | 18.87 |
| K | K series | Yes | 2287.68 | 151.51 | 2.37 |
| K | L series | No | -4559.48 | 317.07 | 7.73 |
| Ca | K series | Yes | 545.70 | 134.69 | 0.64 |
| Ca | L series | No | -3016.30 | 290.96 | 10.17 |
| Au | L series | No | 17641.58 | 371.40 | 1.16 |
| Au | M series | No | 38259.29 | 646.97 | 5.42 |
|  | Noise 1 | No | 137930.29 | 2566.83 | 24.11 |
|  | Noise 2 | No | -158509.91 | 4671.28 | 23.23 |
|  | Noise 3 | No | 90043.67 | 2448.35 | 24.06 |

| Label: | Map Sum Spectrum |
| --- | --- |
| Element List Type: | Current Spectrum |
| Processing Option: | All Elements |
| Specimen Coating: | On |
| Beam Calibration Element Coating: | Off |
| Coating Element: | Gold |
| Coating Thickness: | 12 nm |
| Coating Density: | 19.32 g/cm³ |
| Automatic Line Selection: | Enabled |
| Normalization: | Enabled |
| Thresholding: | Sigma level = 2 |
| Detector Window Correction: | Disabled |
| Deconvolution Elements: | None |
| Selected Standards: | Quant Standardizations [ Factory ] |
| Pulse Pile Up Correction: | Succeeded |
| Detector file: | X-Max 7 |
| Efficiency: | File based |
