# Supplementary material for: Sporosarcina pasteurii can clog and strengthen a porous medium mimic
Source: PLoS One. 2018 Nov 30;13(11):e0207489. doi: 10.1371/journal.pone.0207489 (PMC6267956; doi:10.1371/journal.pone.0207489)
Supplement: S1 Dataset — (ZIP) [file pone.0207489.s002.zip › Raw Data/(for Fig. 5) EDX/negative/Project 3_Site 1_2017-06-01_18-52-48.docx]

6/1/2017 6:45:21 PM

Specimen 1


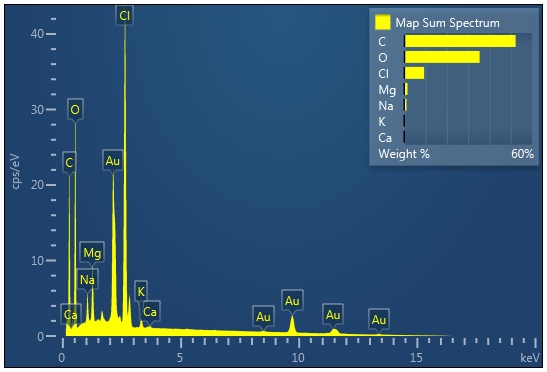


| Element | Line Type | Apparent Concentration | k Ratio | Wt% | Wt% Sigma | Standard Label | Factory Standard | Standard Calibration Date |
| --- | --- | --- | --- | --- | --- | --- | --- | --- |
| C | K series | 21.14 | 0.21142 | 52.32 | 0.13 | C Vit | Yes |  |
| O | K series | 39.41 | 0.13262 | 35.41 | 0.11 | SiO2 | Yes |  |
| Na | K series | 2.77 | 0.01171 | 1.04 | 0.01 | Albite | Yes |  |
| Mg | K series | 3.75 | 0.02488 | 1.50 | 0.01 | MgO | Yes |  |
| Cl | K series | 29.47 | 0.25754 | 9.32 | 0.03 | NaCl | Yes |  |
| K | K series | 1.13 | 0.00956 | 0.34 | 0.01 | KBr | Yes |  |
| Ca | K series | 0.24 | 0.00213 | 0.07 | 0.01 | Wollastonite | Yes |  |
| Total: |  |  |  | 100.00 |  |  |  |  |

| Element | Line Type | Quant | Area | Sigma | Fit Index |
| --- | --- | --- | --- | --- | --- |
| C | K series | Yes | 260293.97 | 1256.90 | 74.21 |
| O | K series | Yes | 336467.66 | 1039.89 | 186.87 |
| Na | K series | Yes | 49328.98 | 608.88 | 2.02 |
| Mg | K series | Yes | 107815.42 | 743.59 | 9.51 |
| Cl | K series | Yes | 882734.14 | 1665.49 | 54.69 |
| Cl | L series | No | 5701.23 | 472.56 | 40.65 |
| K | K series | Yes | 26947.69 | 554.44 | 8.62 |
| K | L series | No | -76832.05 | 1285.44 | 63.06 |
| Ca | K series | Yes | 5364.69 | 490.52 | 1.11 |
| Ca | L series | No | -42834.75 | 1144.36 | 159.81 |
| Au | L series | No | 136926.38 | 1020.94 | 3.47 |
| Au | M series | No | 595784.56 | 2520.28 | 47.31 |
|  | Noise 1 | No | 121068.76 | 2937.27 | 16.62 |
|  | Noise 2 | No | -136555.69 | 5272.92 | 16.03 |
|  | Noise 3 | No | 81890.16 | 2738.22 | 16.50 |

| Label: | Map Sum Spectrum |
| --- | --- |
| Element List Type: | Current Spectrum |
| Processing Option: | All Elements |
| Specimen Coating: | On |
| Beam Calibration Element Coating: | Off |
| Coating Element: | Gold |
| Coating Thickness: | 12 nm |
| Coating Density: | 19.32 g/cm³ |
| Automatic Line Selection: | Enabled |
| Normalization: | Enabled |
| Thresholding: | Sigma level = 2 |
| Detector Window Correction: | Disabled |
| Deconvolution Elements: | None |
| Selected Standards: | Quant Standardizations [ Factory ] |
| Pulse Pile Up Correction: | Succeeded |
| Detector file: | X-Max 7 |
| Efficiency: | File based |
