# Supplementary material for: Sporosarcina pasteurii can clog and strengthen a porous medium mimic
Source: PLoS One. 2018 Nov 30;13(11):e0207489. doi: 10.1371/journal.pone.0207489 (PMC6267956; doi:10.1371/journal.pone.0207489)
Supplement: S1 Dataset — (ZIP) [file pone.0207489.s002.zip › Raw Data/(for Fig. 5) EDX/positive/Project 1_Site 1_2017-05-19_11-12-18.docx]

5/19/2017 11:07:12 AM

Specimen 1


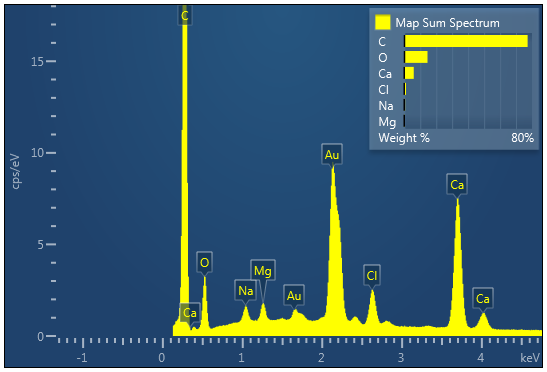


| Element | Line Type | Apparent Concentration | k Ratio | Wt% | Wt% Sigma | Standard Label | Factory Standard | Standard Calibration Date |
| --- | --- | --- | --- | --- | --- | --- | --- | --- |
| C | K series | 32.79 | 0.32793 | 77.28 | 0.14 | C Vit | Yes |  |
| O | K series | 4.16 | 0.01399 | 14.59 | 0.14 | SiO2 | Yes |  |
| Na | K series | 0.61 | 0.00258 | 0.55 | 0.02 | Albite | Yes |  |
| Mg | K series | 0.49 | 0.00324 | 0.48 | 0.01 | MgO | Yes |  |
| Cl | K series | 1.36 | 0.01191 | 1.13 | 0.01 | NaCl | Yes |  |
| Ca | K series | 7.57 | 0.06764 | 5.98 | 0.03 | Wollastonite | Yes |  |
| Total: |  |  |  | 100.00 |  |  |  |  |

| Element | Line Type | Quant | Area | Sigma | Fit Index |
| --- | --- | --- | --- | --- | --- |
| C | K series | Yes | 403731.58 | 1112.40 | 1514.22 |
| Cl | K series | Yes | 40822.53 | 485.52 | 13.40 |
| Cl | L series | No | 11894.14 | 322.95 | 1457.02 |
| Ca | K series | Yes | 170552.70 | 742.07 | 4.22 |
| Ca | L series | No | -3352.91 | 793.01 | 1162.57 |
| Na | K series | Yes | 10850.20 | 356.35 | 5.65 |
| Mg | K series | Yes | 14025.23 | 377.70 | 14.02 |
| O | K series | Yes | 35502.21 | 388.82 | 326.82 |
| Au | L series | No | 55714.13 | 596.02 | 5.53 |
| Au | M series | No | 262554.66 | 1624.96 | 18.72 |
|  | Noise 1 | No | 148417.51 | 3065.60 | 56.27 |
|  | Noise 2 | No | -173997.32 | 5509.53 | 54.28 |
|  | Noise 3 | No | 98041.61 | 2861.82 | 53.97 |

| Label: | Map Sum Spectrum |
| --- | --- |
| Element List Type: | Current Spectrum |
| Processing Option: | All Elements |
| Specimen Coating: | On |
| Beam Calibration Element Coating: | Off |
| Coating Element: | Gold |
| Coating Thickness: | 12 nm |
| Coating Density: | 19.32 g/cm³ |
| Automatic Line Selection: | Enabled |
| Normalization: | Enabled |
| Thresholding: | Sigma level = 2 |
| Detector Window Correction: | Disabled |
| Deconvolution Elements: | None |
| Selected Standards: | Quant Standardizations [ Factory ] |
| Pulse Pile Up Correction: | Succeeded |
| Detector file: | X-Max 7 |
| Efficiency: | File based |
