# Supplementary material for: Sporosarcina pasteurii can clog and strengthen a porous medium mimic
Source: PLoS One. 2018 Nov 30;13(11):e0207489. doi: 10.1371/journal.pone.0207489 (PMC6267956; doi:10.1371/journal.pone.0207489)
Supplement: S1 Dataset — (ZIP) [file pone.0207489.s002.zip › Raw Data/(for Fig. 5) EDX/positive/Project 1_Site 3_2017-05-19_11-47-54.docx]

5/19/2017 11:26:23 AM

Specimen 1


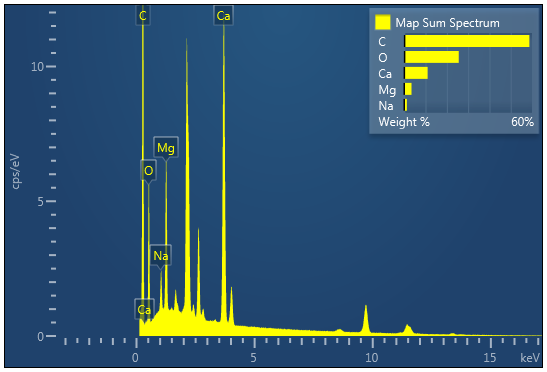


| Element | Line Type | Apparent Concentration | k Ratio | Wt% | Wt% Sigma | Standard Label | Factory Standard | Standard Calibration Date |
| --- | --- | --- | --- | --- | --- | --- | --- | --- |
| C | K series | 18.66 | 0.18661 | 58.76 | 0.15 | C Vit | Yes |  |
| O | K series | 7.35 | 0.02475 | 25.56 | 0.16 | SiO2 | Yes |  |
| Na | K series | 1.12 | 0.00474 | 1.25 | 0.03 | Albite | Yes |  |
| Mg | K series | 2.86 | 0.01899 | 3.43 | 0.03 | MgO | Yes |  |
| Ca | K series | 12.30 | 0.10994 | 11.00 | 0.05 | Wollastonite | Yes |  |
| Total: |  |  |  | 100.00 |  |  |  |  |

| Element | Line Type | Quant | Area | Sigma | Fit Index |
| --- | --- | --- | --- | --- | --- |
| C | K series | Yes | 229747.62 | 890.59 | 860.58 |
| O | K series | Yes | 62790.98 | 493.07 | 187.96 |
| Na | K series | Yes | 19955.21 | 416.82 | 3.60 |
| Mg | K series | Yes | 82265.13 | 594.84 | 8.74 |
| Ca | K series | Yes | 277210.51 | 911.27 | 6.67 |
| Ca | L series | No | -5613.27 | 801.36 | 679.87 |
| Au | L series | No | 61632.84 | 630.37 | 4.70 |
| Au | M series | No | 308486.58 | 1724.68 | 908.16 |
|  | Noise 1 | No | 130271.46 | 2863.93 | 14.44 |
|  | Noise 2 | No | -147771.86 | 5150.02 | 13.92 |
|  | Noise 3 | No | 84389.92 | 2674.28 | 12.95 |

| Label: | Map Sum Spectrum |
| --- | --- |
| Element List Type: | Current Spectrum |
| Processing Option: | All Elements |
| Specimen Coating: | On |
| Beam Calibration Element Coating: | Off |
| Coating Element: | Gold |
| Coating Thickness: | 12 nm |
| Coating Density: | 19.32 g/cm³ |
| Automatic Line Selection: | Enabled |
| Normalization: | Enabled |
| Thresholding: | Sigma level = 2 |
| Detector Window Correction: | Disabled |
| Deconvolution Elements: | None |
| Selected Standards: | Quant Standardizations [ Factory ] |
| Pulse Pile Up Correction: | Succeeded |
| Detector file: | X-Max 7 |
| Efficiency: | File based |
