# Supplementary material for: Sporosarcina pasteurii can clog and strengthen a porous medium mimic
Source: PLoS One. 2018 Nov 30;13(11):e0207489. doi: 10.1371/journal.pone.0207489 (PMC6267956; doi:10.1371/journal.pone.0207489)
Supplement: S1 Dataset — (ZIP) [file pone.0207489.s002.zip › Raw Data/(for Fig. 5) EDX/positive/Project 1_Site 4_2017-05-19_11-58-53.docx]

5/19/2017 11:56:16 AM


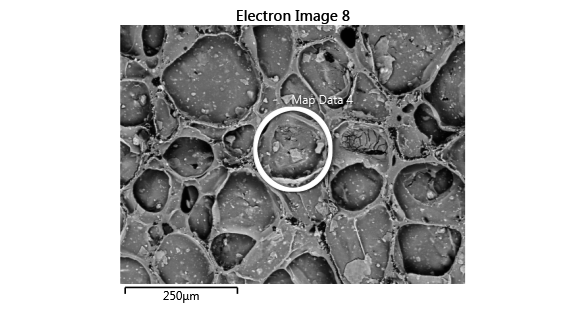


| Label: | Electron Image 8 |
| --- | --- |
| Collected: | 5/19/2017 11:50:29 AM |
| Input Signal: | BSE |
| Resolution (Width): | 1024 pixels |
| Resolution (Height): | 768 pixels |
| Image Width: | 770μm |
| Image Height: | 577μm |
| Stage Tilt Degrees: | 0.00° |
| Specimen Tilt Degrees: | 0.00° |
| Software Tilt Correction: | Not applied |
| Magnification: | 153 x |
| Number of Averaged Frames: | 1 |
| Dwell Time: | 20μs |


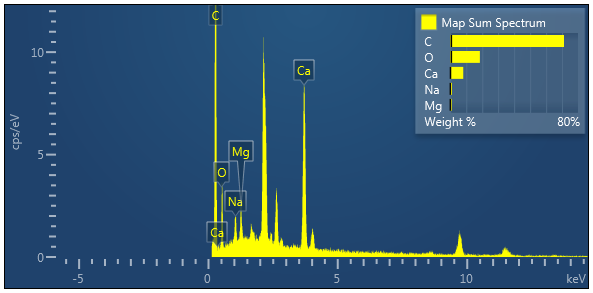


| Label: | Map Sum Spectrum |
| --- | --- |
| Element List Type: | Current Spectrum |
| Processing Option: | All Elements |
| Specimen Coating: | On |
| Beam Calibration Element Coating: | Off |
| Coating Element: | Gold |
| Coating Thickness: | 12 nm |
| Coating Density: | 19.32 g/cm³ |
| Automatic Line Selection: | Enabled |
| Normalization: | Enabled |
| Thresholding: | Sigma level = 2 |
| Detector Window Correction: | Disabled |
| Deconvolution Elements: | None |
| Selected Standards: | Quant Standardizations [ Factory ] |
| Pulse Pile Up Correction: | Succeeded |
| Detector file: | X-Max 7 |
| Efficiency: | File based |

| Element | Line Type | Apparent Concentration | k Ratio | Wt% | Wt% Sigma | Standard Label | Factory Standard | Standard Calibration Date |
| --- | --- | --- | --- | --- | --- | --- | --- | --- |
| C | K series | 26.53 | 0.26526 | 71.20 | 0.67 | C Vit | Yes |  |
| O | K series | 4.55 | 0.01531 | 18.57 | 0.70 | SiO2 | Yes |  |
| Na | K series | 0.97 | 0.00410 | 1.11 | 0.11 | Albite | Yes |  |
| Mg | K series | 0.78 | 0.00520 | 0.97 | 0.08 | MgO | Yes |  |
| Ca | K series | 8.54 | 0.07631 | 8.16 | 0.17 | Wollastonite | Yes |  |
| Total: |  |  |  | 100.00 |  |  |  |  |
