# Supplementary material for: Sporosarcina pasteurii can clog and strengthen a porous medium mimic
Source: PLoS One. 2018 Nov 30;13(11):e0207489. doi: 10.1371/journal.pone.0207489 (PMC6267956; doi:10.1371/journal.pone.0207489)

Specimen 1

Click here to enter text.


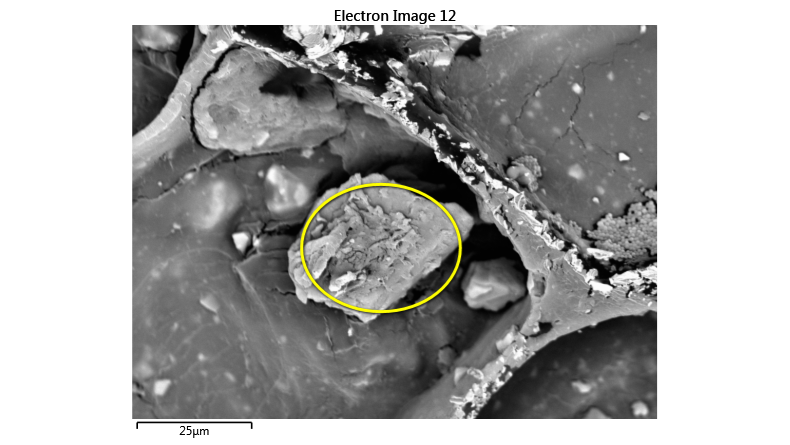


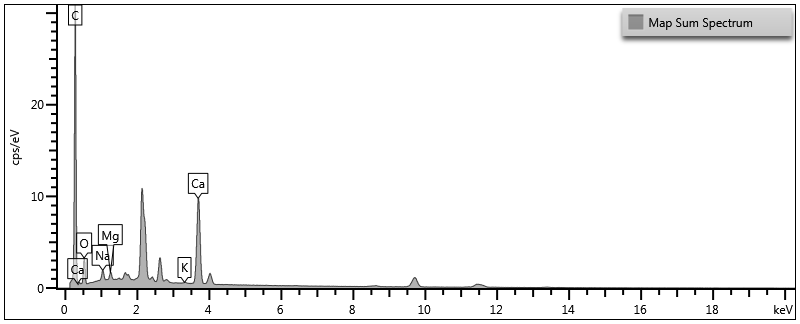


| Element | Line Type | Apparent Concentration | k Ratio | Wt% | Wt% Sigma | Standard Label | Factory Standard | Standard Calibration Date |
| --- | --- | --- | --- | --- | --- | --- | --- | --- |
| C | K series | 31.83 | 0.31831 | 73.43 | 0.15 | C Vit | Yes |  |
| O | K series | 4.26 | 0.01434 | 16.48 | 0.16 | SiO2 | Yes |  |
| Na | K series | 0.80 | 0.00336 | 0.82 | 0.02 | Albite | Yes |  |
| Mg | K series | 0.42 | 0.00276 | 0.46 | 0.01 | MgO | Yes |  |
| K | K series | 0.08 | 0.00072 | 0.07 | 0.01 | KBr | Yes |  |
| Ca | K series | 10.06 | 0.08987 | 8.73 | 0.04 | Wollastonite | Yes |  |
| Total: |  |  |  | 100.00 |  |  |  |  |

| Element | Line Type | Quant | Area | Sigma | Fit Index |
| --- | --- | --- | --- | --- | --- |
| C | K series | Yes | 391885.65 | 1338.71 | 122.26 |
| Ca | K series | Yes | 226608.71 | 835.51 | 2.64 |
| Ca | L series | No | -10508.96 | 819.43 | 103.98 |
| O | K series | Yes | 36373.86 | 399.74 | 52.83 |
| Na | K series | Yes | 14154.09 | 379.22 | 6.60 |
| Mg | K series | Yes | 11979.85 | 380.20 | 24.13 |
| K | K series | Yes | 2025.35 | 333.15 | 519.03 |
| K | L series | No | -100407.25 | 1194.67 | 126.80 |
| Au | L series | No | 61611.76 | 626.30 | 5.42 |
| Au | M series | No | 302090.05 | 1735.02 | 659.54 |
|  | Noise 1 | No | 213634.89 | 3507.52 | 41.34 |
|  | Noise 2 | No | -262569.61 | 6328.08 | 40.04 |
|  | Noise 3 | No | 142784.37 | 3300.50 | 40.46 |

| Label: | Map Sum Spectrum |
| --- | --- |
| Source: | Acquired |
| Created: | 5/19/2017 12:13:52 PM |
| Livetime: | 195.0s |
| Process Time: | 5 |
| Accelerating Voltage: | 20.00kV |
| Magnification: | 1026 x |
| Working Distance: | 8.3mm |
| Specimen Tilt (degrees): | 0.0 |
| Elevation (degrees): | 35.0 |
| Azimuth (degrees): | 0.0 |
| Number Of Channels: | 2048 |
| Energy Range (keV): | 20 keV |
| Energy per Channel (eV): | 10.0eV |
| Detector Type Id: | 28 |
| Detector Type: | X-Max |
| Window Type: | SATW |
| Pulse Pile Up Correction: | Succeeded |
| Primary Detector: | EDS 1 |
| Primary Detector Serial Number: | 37456 |


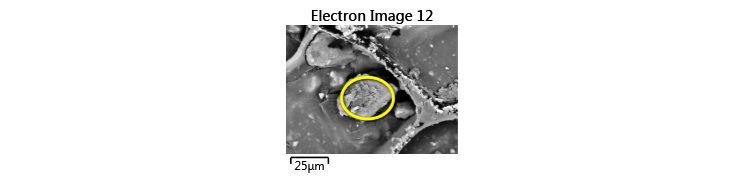

Supplement: S1 Dataset — (ZIP) [file pone.0207489.s002.zip › Raw Data/(for Fig. 5) EDX/positive/Project 1_Site 7_2017-05-19_12-21-58.docx]
