# Supplementary material for: Sporosarcina pasteurii can clog and strengthen a porous medium mimic
Source: PLoS One. 2018 Nov 30;13(11):e0207489. doi: 10.1371/journal.pone.0207489 (PMC6267956; doi:10.1371/journal.pone.0207489)

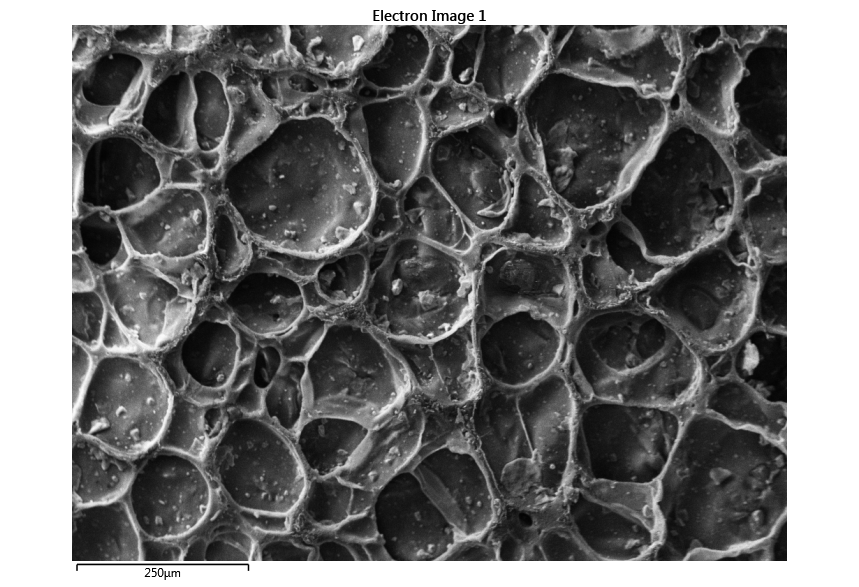


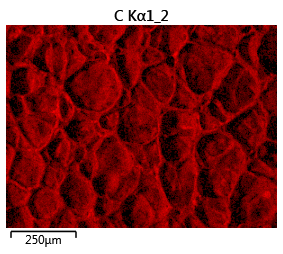

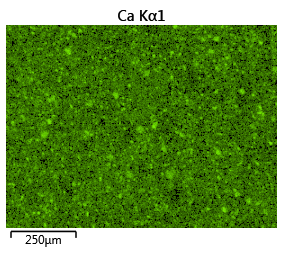

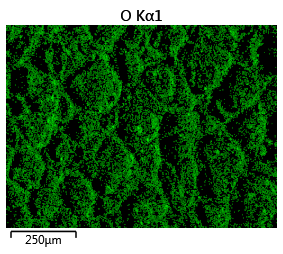


| Element | Line Type | Apparent Concentration | k Ratio | Wt% | Wt% Sigma | Standard Label | Factory Standard | Standard Calibration Date |
| --- | --- | --- | --- | --- | --- | --- | --- | --- |
| C | K series | 32.79 | 0.32793 | 77.28 | 0.14 | C Vit | Yes |  |
| Ca | K series | 7.57 | 0.06764 | 5.98 | 0.03 | Wollastonite | Yes |  |
| O | K series | 4.16 | 0.01399 | 14.59 | 0.14 | SiO2 | Yes |  |
| Cl | K series | 1.36 | 0.01191 | 1.13 | 0.01 | NaCl | Yes |  |
| Na | K series | 0.61 | 0.00258 | 0.55 | 0.02 | Albite | Yes |  |
| Mg | K series | 0.49 | 0.00324 | 0.48 | 0.01 | MgO | Yes |  |
| Total: |  |  |  | 100.00 |  |  |  |  |

| Element | Line Type | Quant | Area | Sigma | Fit Index |
| --- | --- | --- | --- | --- | --- |
| C | K series | Yes | 403731.58 | 1112.40 | 1514.22 |
| Ca | K series | Yes | 170552.70 | 742.07 | 4.22 |
| Ca | L series | No | -3352.91 | 793.01 | 1162.57 |
| O | K series | Yes | 35502.21 | 388.82 | 326.82 |
| Au | L series | No | 55714.13 | 596.02 | 5.53 |
| Au | M series | No | 262554.66 | 1624.96 | 18.72 |
|  | Noise 1 | No | 148417.51 | 3065.60 | 56.27 |
|  | Noise 2 | No | -173997.32 | 5509.53 | 54.28 |
|  | Noise 3 | No | 98041.61 | 2861.82 | 53.97 |

Specimen 1

Click here to enter text.


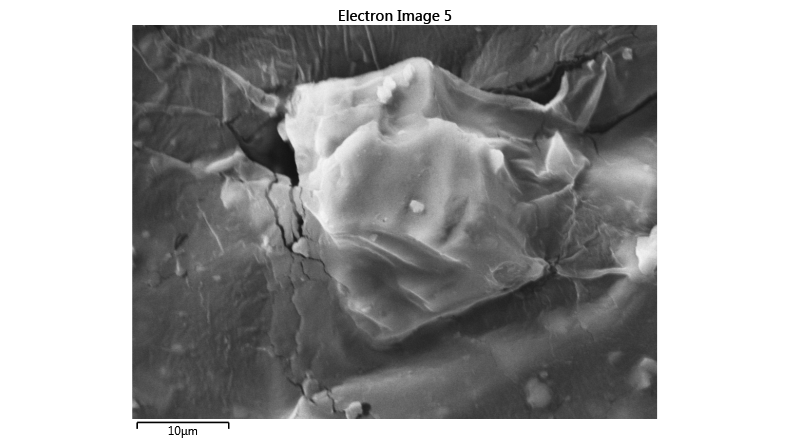


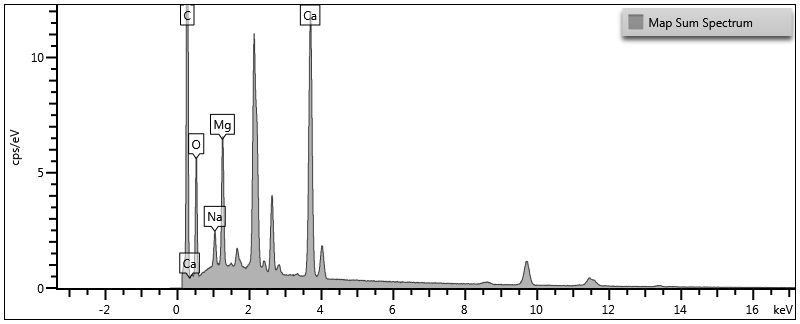


| Element | Line Type | Apparent Concentration | k Ratio | Wt% | Wt% Sigma | Standard Label | Factory Standard | Standard Calibration Date |
| --- | --- | --- | --- | --- | --- | --- | --- | --- |
| C | K series | 18.66 | 0.18661 | 58.76 | 0.15 | C Vit | Yes |  |
| O | K series | 7.35 | 0.02475 | 25.56 | 0.16 | SiO2 | Yes |  |
| Ca | K series | 12.30 | 0.10994 | 11.00 | 0.05 | Wollastonite | Yes |  |
| Total: |  |  |  | 100.00 |  |  |  |  |

| Element | Line Type | Quant | Area | Sigma | Fit Index |
| --- | --- | --- | --- | --- | --- |
| C | K series | Yes | 229747.62 | 890.59 | 860.58 |
| O | K series | Yes | 62790.98 | 493.07 | 187.96 |
| Ca | K series | Yes | 277210.51 | 911.27 | 6.67 |
| Ca | L series | No | -5613.27 | 801.36 | 679.87 |
| Au | L series | No | 61632.84 | 630.37 | 4.70 |
| Au | M series | No | 308486.58 | 1724.68 | 908.16 |
|  | Noise 1 | No | 130271.46 | 2863.93 | 14.44 |
|  | Noise 2 | No | -147771.86 | 5150.02 | 13.92 |
|  | Noise 3 | No | 84389.92 | 2674.28 | 12.95 |

| Label: | Map Sum Spectrum |
| --- | --- |
| Source: | Acquired |
| Created: | 5/19/2017 11:26:23 AM |
| Livetime: | 195.0s |
| Process Time: | 5 |
| Accelerating Voltage: | 20.00kV |
| Magnification: | 2052 x |
| Working Distance: | 8.4mm |
| Specimen Tilt (degrees): | 0.0 |
| Elevation (degrees): | 35.0 |
| Azimuth (degrees): | 0.0 |
| Number Of Channels: | 2048 |
| Energy Range (keV): | 20 keV |
| Energy per Channel (eV): | 10.0eV |
| Detector Type Id: | 28 |
| Detector Type: | X-Max |
| Window Type: | SATW |
| Pulse Pile Up Correction: | Succeeded |
| Primary Detector: | EDS 1 |
| Primary Detector Serial Number: | 37456 |


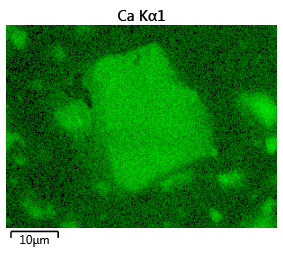

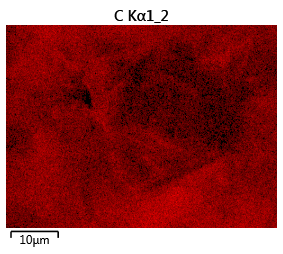

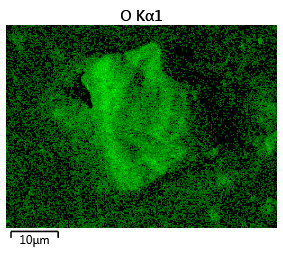


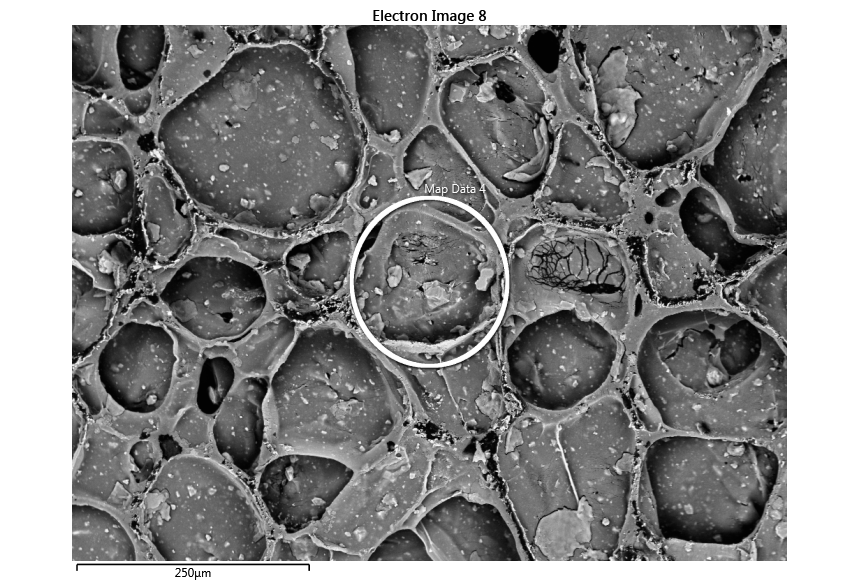


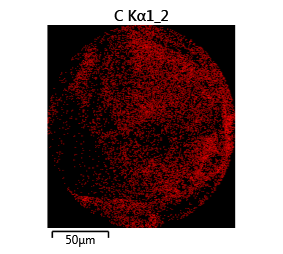

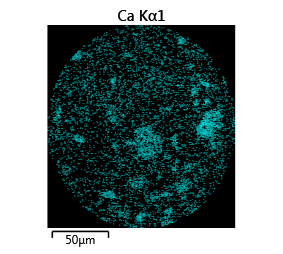

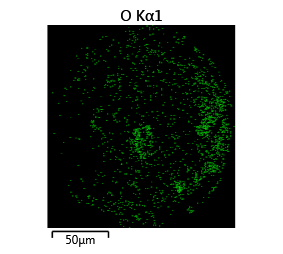


5/19/2017 11:56:16 AM

| Label: | Electron Image 8 |
| --- | --- |
| Collected: | 5/19/2017 11:50:29 AM |
| Input Signal: | BSE |
| Resolution (Width): | 1024 pixels |
| Resolution (Height): | 768 pixels |
| Image Width: | 770μm |
| Image Height: | 577μm |
| Stage Tilt Degrees: | 0.00° |
| Specimen Tilt Degrees: | 0.00° |
| Software Tilt Correction: | Not applied |
| Magnification: | 153 x |
| Number of Averaged Frames: | 1 |
| Dwell Time: | 20μs |


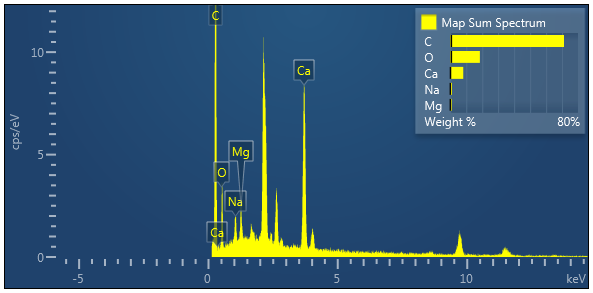


| Label: | Map Sum Spectrum |  |  |  |  |  |  |  |
| --- | --- | --- | --- | --- | --- | --- | --- | --- |
| Element List Type: | Current Spectrum |  |  |  |  |  |  |  |
| Processing Option: | All Elements |  |  |  |  |  |  |  |
| Specimen Coating: | On |  |  |  |  |  |  |  |
| Beam Calibration Element Coating: | Off |  |  |  |  |  |  |  |
| Coating Element: | Gold |  |  |  |  |  |  |  |
| Coating Thickness: | 12 nm |  |  |  |  |  |  |  |
| Coating Density: | 19.32 g/cm³ |  |  |  |  |  |  |  |
| Automatic Line Selection: | Enabled |  |  |  |  |  |  |  |
| Normalization: | Enabled |  |  |  |  |  |  |  |
| Thresholding: | Sigma level = 2 |  |  |  |  |  |  |  |
| Detector Window Correction: | Disabled |  |  |  |  |  |  |  |
| Deconvolution Elements: | None |  |  |  |  |  |  |  |
| Selected Standards: | Quant Standardizations [ Factory ] |  |  |  |  |  |  |  |
| Pulse Pile Up Correction: | Succeeded |  |  |  |  |  |  |  |
| Detector file: | X-Max 7 |  |  |  |  |  |  |  |
| Efficiency: | File based |  |  |  |  |  |  |  |
| Element | Line Type | Apparent Concentration | k Ratio | Wt% | Wt% Sigma | Standard Label | Factory Standard | Standard Calibration Date |
| C | K series | 26.53 | 0.26526 | 71.20 | 0.67 | C Vit | Yes |  |
| O | K series | 4.55 | 0.01531 | 18.57 | 0.70 | SiO2 | Yes |  |
| Ca | K series | 8.54 | 0.07631 | 8.16 | 0.17 | Wollastonite | Yes |  |
| Total: |  |  |  | 100.00 |  |  |  |  |


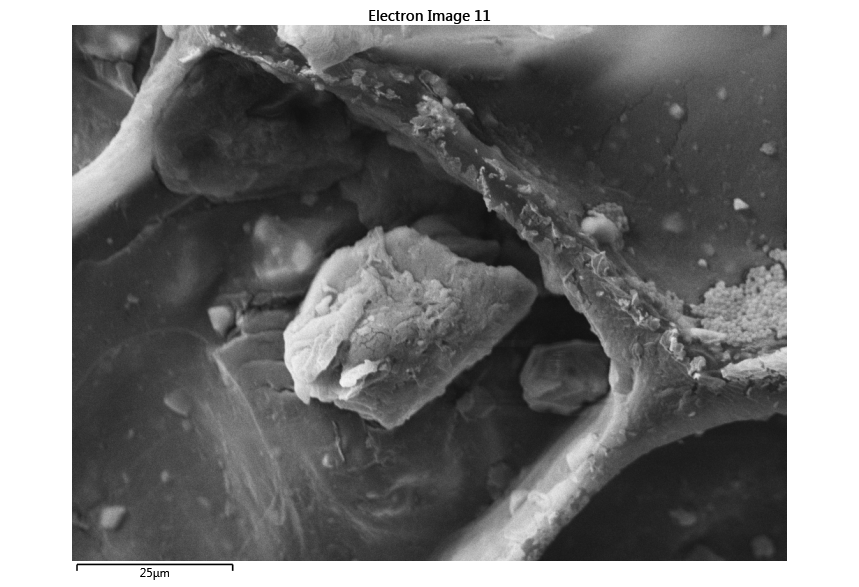


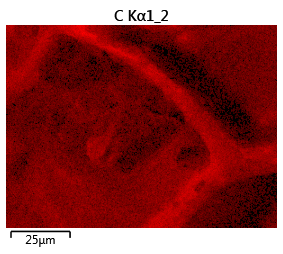

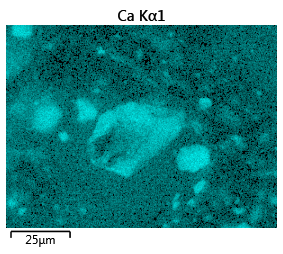

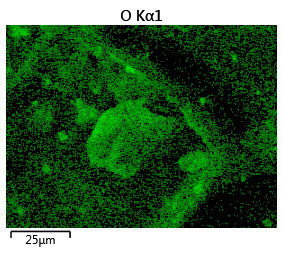


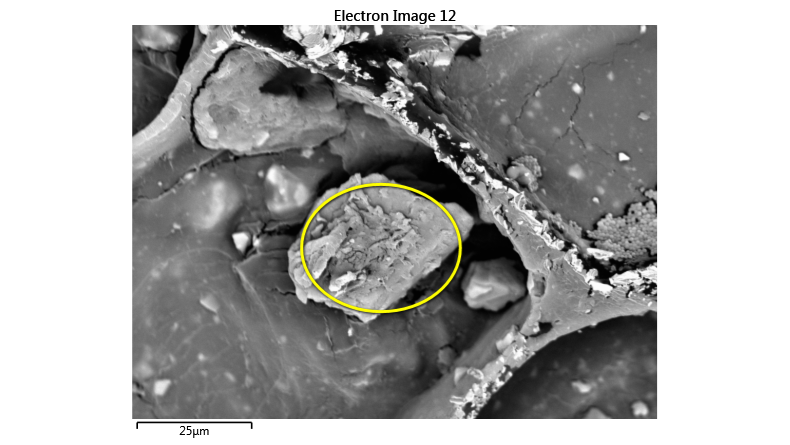


Specimen 1

Click here to enter text.


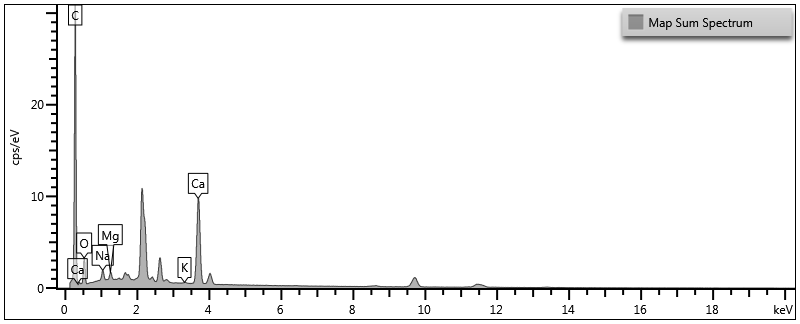


| Element | Line Type | Apparent Concentration | k Ratio | Wt% | Wt% Sigma | Standard Label | Factory Standard | Standard Calibration Date |
| --- | --- | --- | --- | --- | --- | --- | --- | --- |
| C | K series | 31.83 | 0.31831 | 73.43 | 0.15 | C Vit | Yes |  |
| O | K series | 4.26 | 0.01434 | 16.48 | 0.16 | SiO2 | Yes |  |
| Ca | K series | 10.06 | 0.08987 | 8.73 | 0.04 | Wollastonite | Yes |  |
| Na | K series | 0.80 | 0.00336 | 0.82 | 0.02 | Albite | Yes |  |
| Mg | K series | 0.42 | 0.00276 | 0.46 | 0.01 | MgO | Yes |  |
| K | K series | 0.08 | 0.00072 | 0.07 | 0.01 | KBr | Yes |  |
| Total: |  |  |  | 100.00 |  |  |  |  |

| Element | Line Type | Quant | Area | Sigma | Fit Index |
| --- | --- | --- | --- | --- | --- |
| C | K series | Yes | 391885.65 | 1338.71 | 122.26 |
| Ca | K series | Yes | 226608.71 | 835.51 | 2.64 |
| Ca | L series | No | -10508.96 | 819.43 | 103.98 |
| O | K series | Yes | 36373.86 | 399.74 | 52.83 |
| Na | K series | Yes | 14154.09 | 379.22 | 6.60 |
| Mg | K series | Yes | 11979.85 | 380.20 | 24.13 |
| K | K series | Yes | 2025.35 | 333.15 | 519.03 |
| K | L series | No | -100407.25 | 1194.67 | 126.80 |
|  | Noise 1 | No | 213634.89 | 3507.52 | 41.34 |
|  | Noise 2 | No | -262569.61 | 6328.08 | 40.04 |
|  | Noise 3 | No | 142784.37 | 3300.50 | 40.46 |

| Label: | Map Sum Spectrum |
| --- | --- |
| Source: | Acquired |
| Created: | 5/19/2017 12:13:52 PM |
| Livetime: | 195.0s |
| Process Time: | 5 |
| Accelerating Voltage: | 20.00kV |
| Magnification: | 1026 x |
| Working Distance: | 8.3mm |
| Specimen Tilt (degrees): | 0.0 |
| Elevation (degrees): | 35.0 |
| Azimuth (degrees): | 0.0 |
| Number Of Channels: | 2048 |
| Energy Range (keV): | 20 keV |
| Energy per Channel (eV): | 10.0eV |
| Detector Type Id: | 28 |
| Detector Type: | X-Max |
| Window Type: | SATW |
| Pulse Pile Up Correction: | Succeeded |
| Primary Detector: | EDS 1 |
| Primary Detector Serial Number: | 37456 |


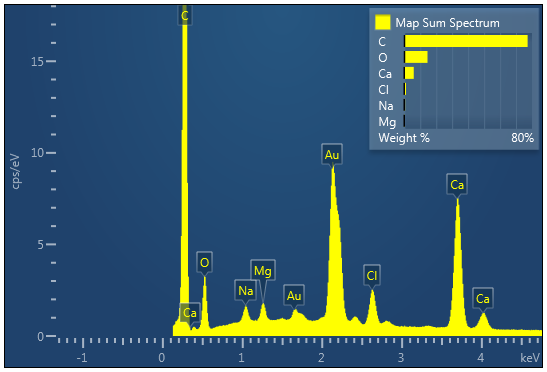

Supplement: S1 Dataset — (ZIP) [file pone.0207489.s002.zip › Raw Data/(for Fig. 5) EDX/positive/Project 3_Site 1_2017-06-01_18-35-50.docx]
