# Supplementary figures and images for: Sporosarcina pasteurii can clog and strengthen a porous medium mimic
Source: PLoS One. 2018 Nov 30;13(11):e0207489. doi: 10.1371/journal.pone.0207489 (PMC6267956; doi:10.1371/journal.pone.0207489)

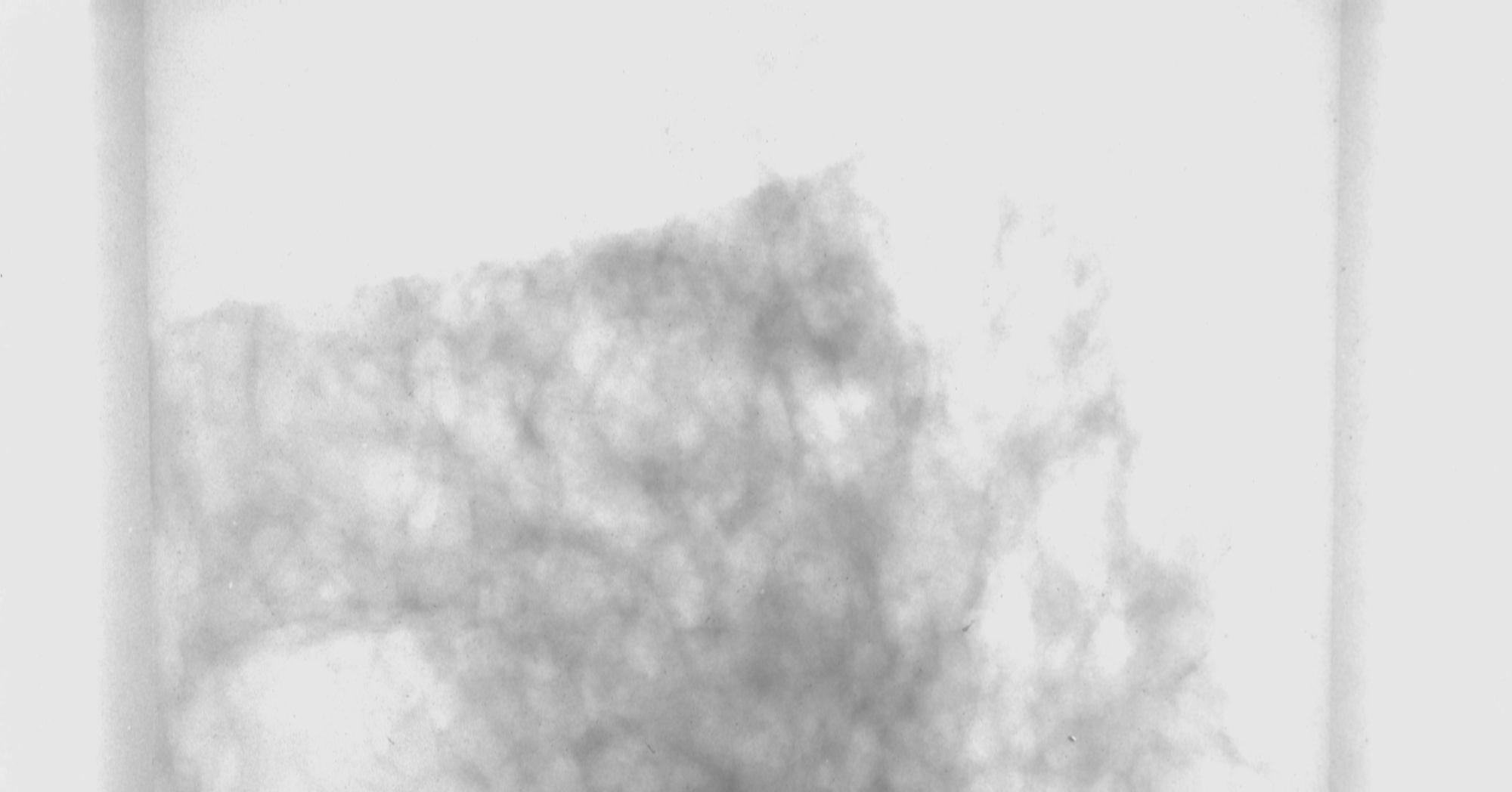

Supplement: S1 Dataset — (ZIP) [file pone.0207489.s002.zip › Raw Data/(for Fig. 4) CT Scans/Clogged/Sponge_clogged_Rec/Sponge_clogged_rec_spr.bmp]

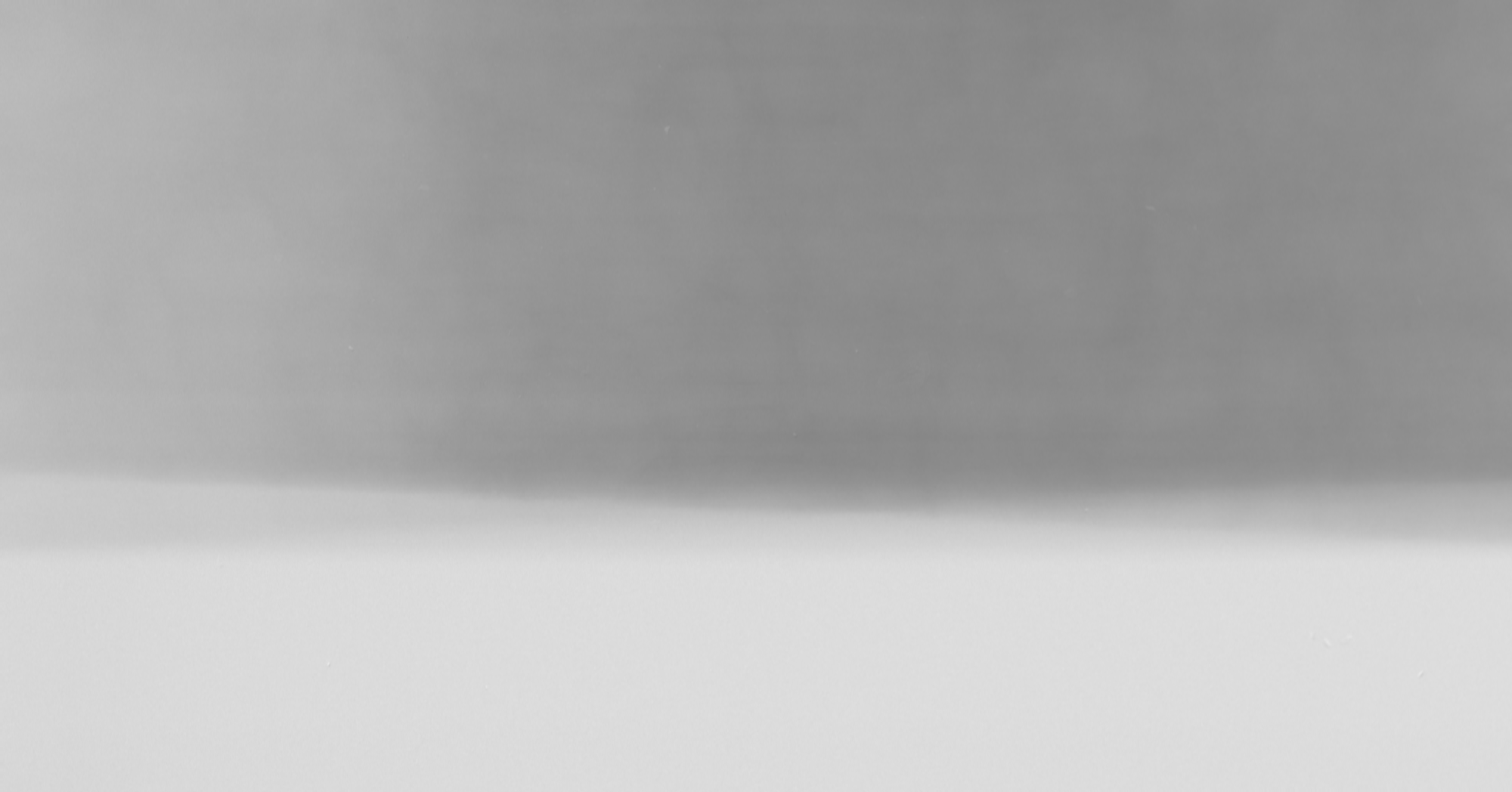

Supplement: S1 Dataset — (ZIP) [file pone.0207489.s002.zip › Raw Data/(for Fig. 4) CT Scans/Unclogged/Sponge_A_arc.tif]

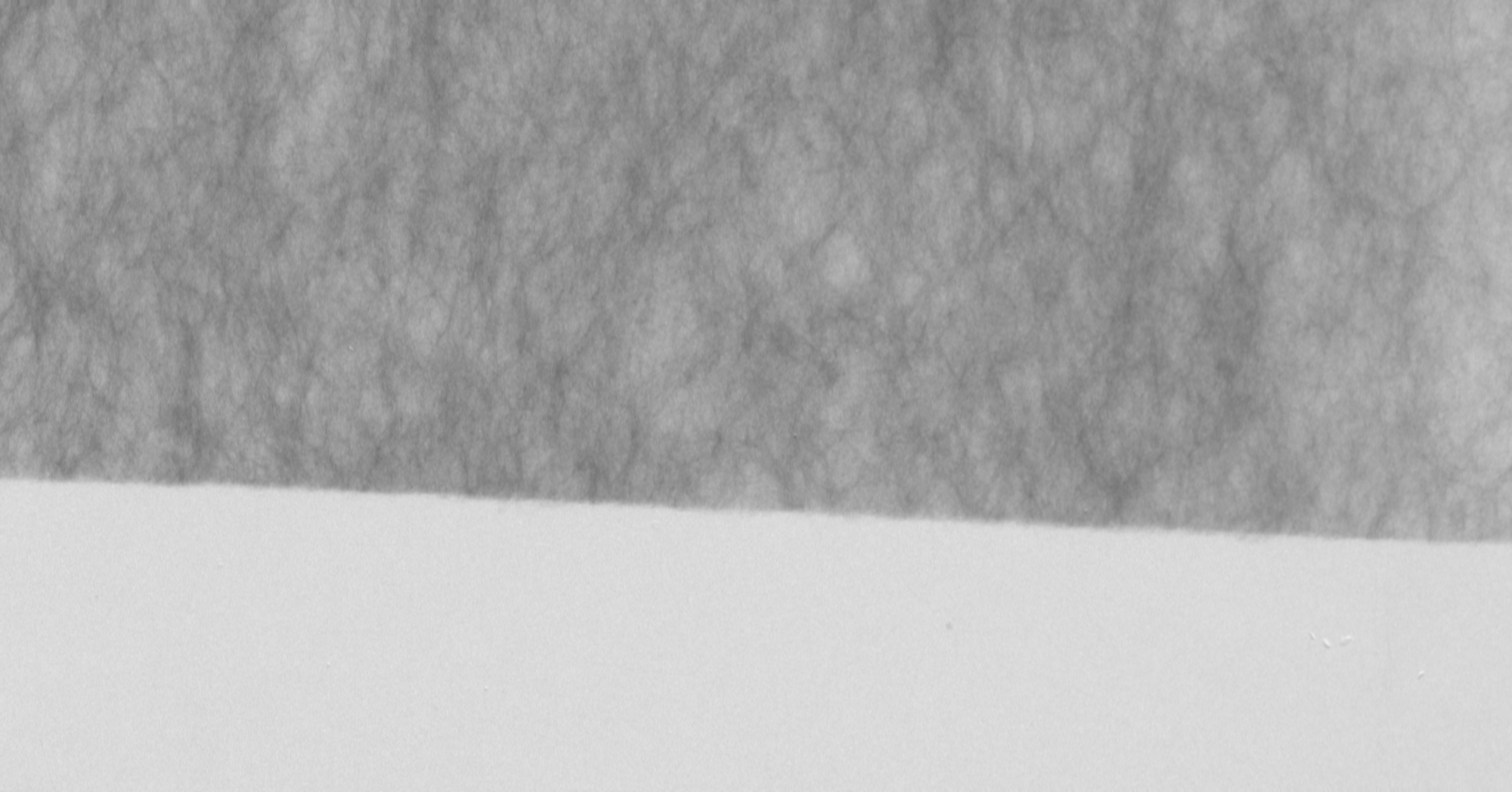

Supplement: S1 Dataset — (ZIP) [file pone.0207489.s002.zip › Raw Data/(for Fig. 4) CT Scans/Unclogged/Sponge_A_Rec/Sponge_A_rec_spr.bmp]

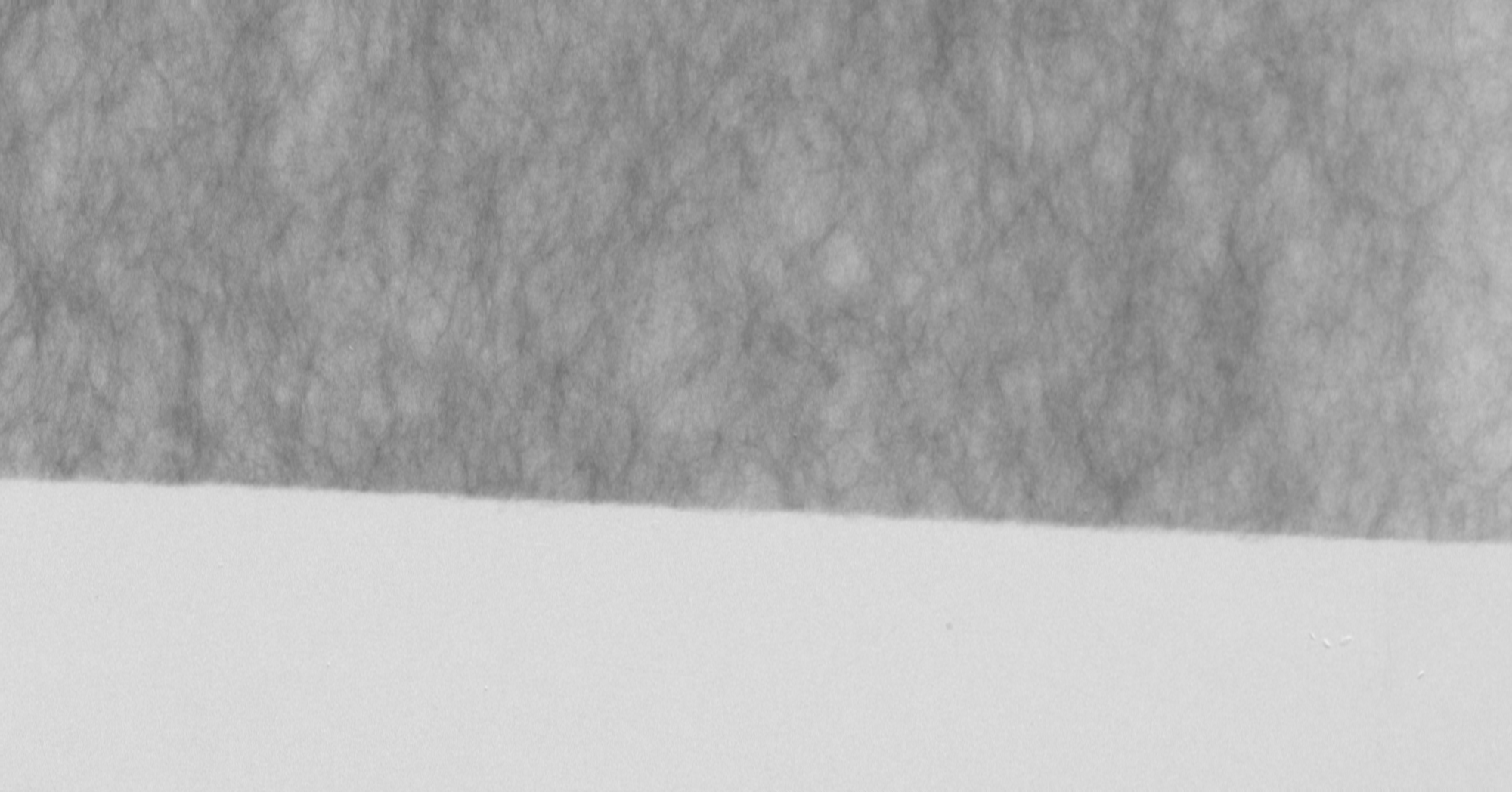

Supplement: S1 Dataset — (ZIP) [file pone.0207489.s002.zip › Raw Data/(for Fig. 4) CT Scans/Unclogged/Sponge_A0000.tif]

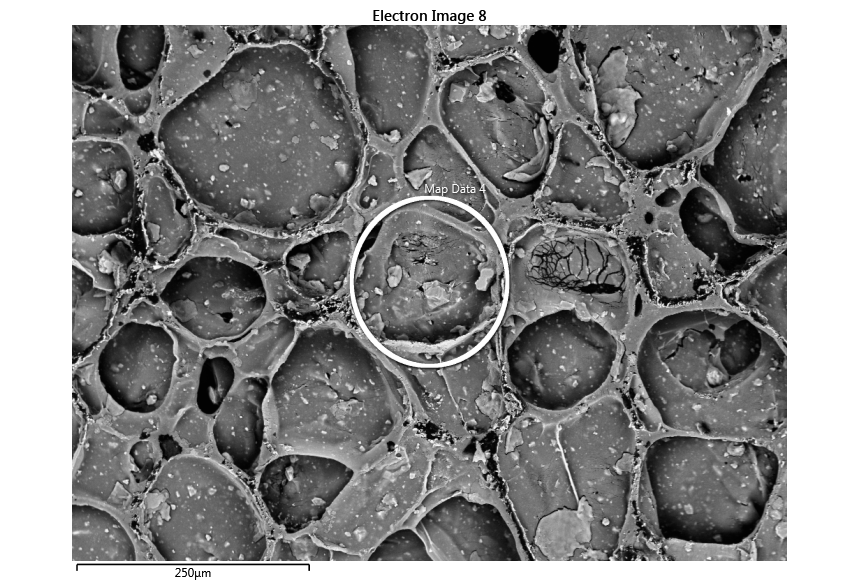


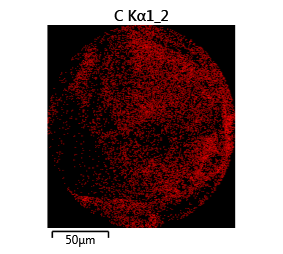

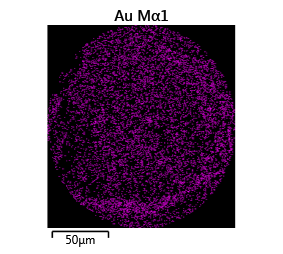

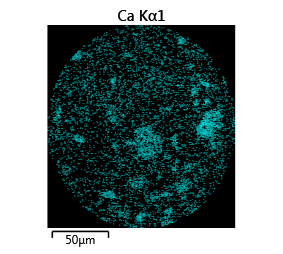

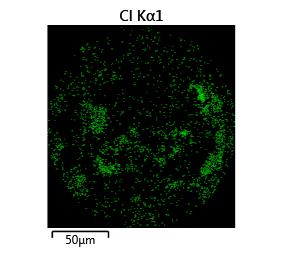

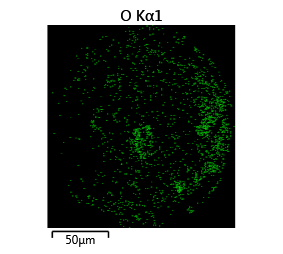

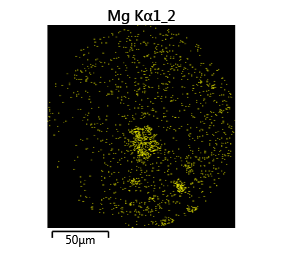

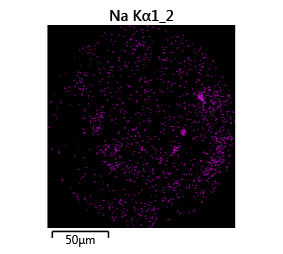

Supplement: S1 Dataset — (ZIP) [file pone.0207489.s002.zip › Raw Data/(for Fig. 5) EDX/positive/Project 1_Site 4_2017-05-19_11-58-21.docx]

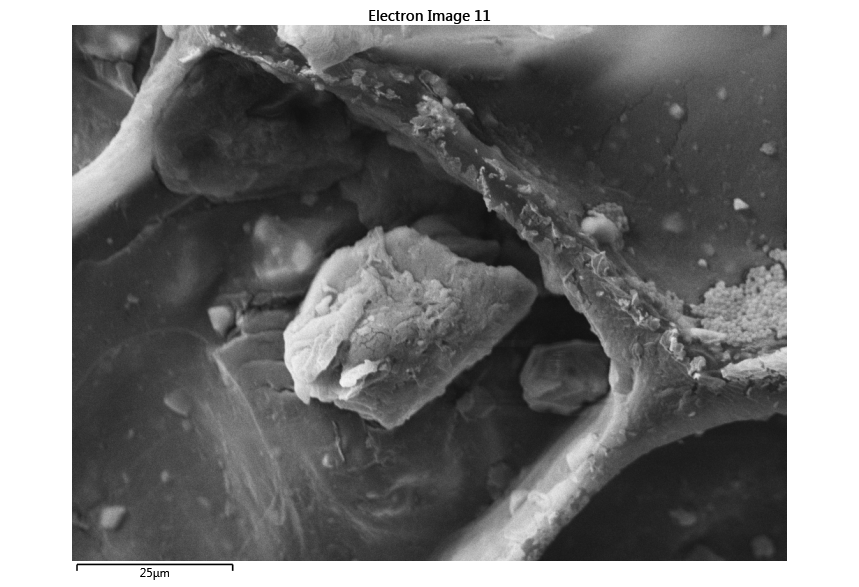


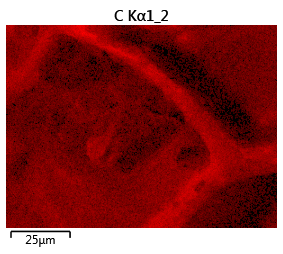

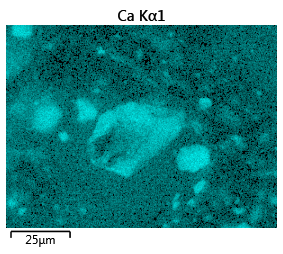

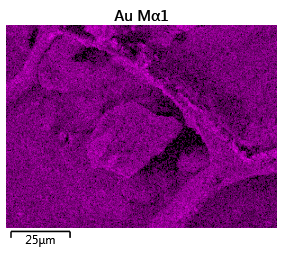

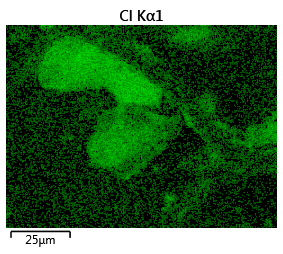

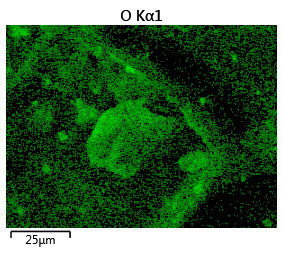

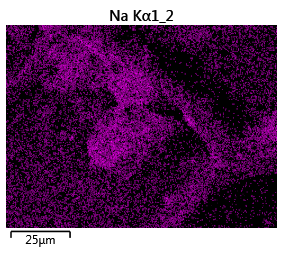

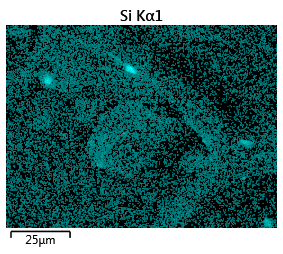

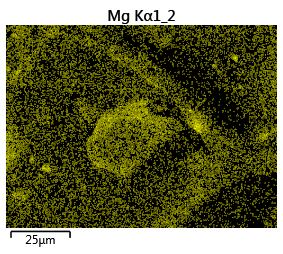

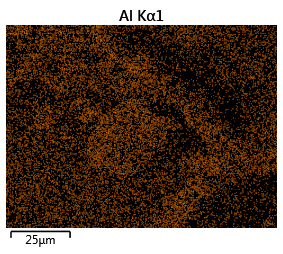

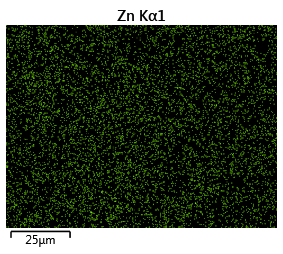

Supplement: S1 Dataset — (ZIP) [file pone.0207489.s002.zip › Raw Data/(for Fig. 5) EDX/positive/Project 1_Site 7_2017-05-19_12-18-46.docx]

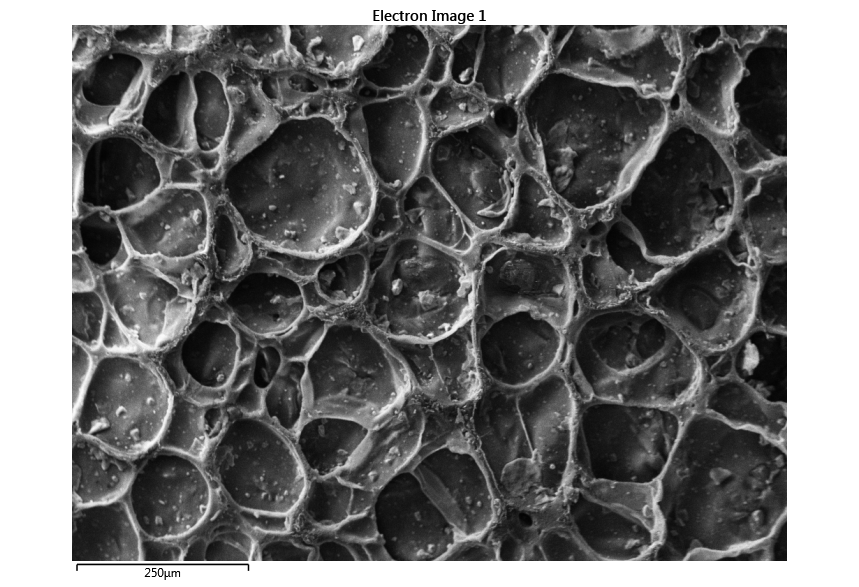


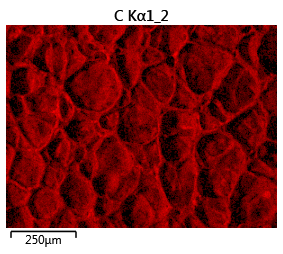

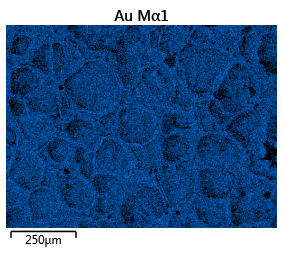

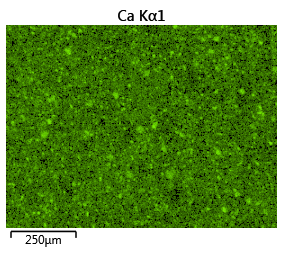

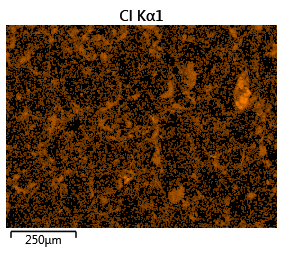

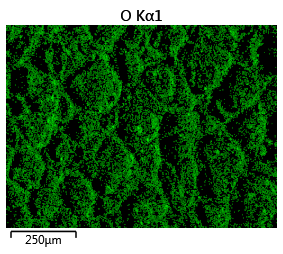

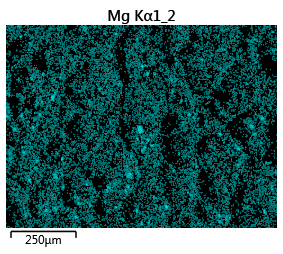

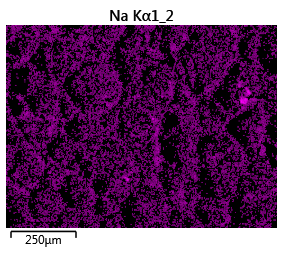

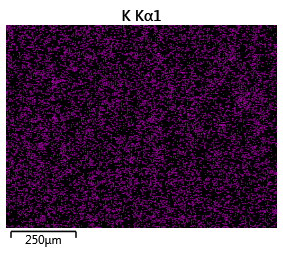

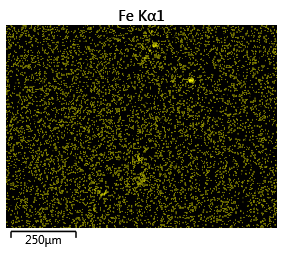

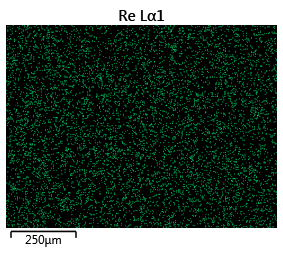

Supplement: S1 Dataset — (ZIP) [file pone.0207489.s002.zip › Raw Data/(for Fig. 5) EDX/positive/Project 2_Site 9_2017-05-19_11-11-50.docx]
